# Block diagonal Calderón preconditioning for scattering at multi-screens

**DOI:** 10.1007/s10543-024-01034-9

**Published:** 2024-09-03

**Authors:** Kristof Cools, Carolina Urzúa-Torres

**Affiliations:** 1 Tech Lane 126, 9052 Ghent, Belgium; 2 Mekelweg 6, 2628 CD Delft, The Netherlands

**Keywords:** Preconditioning, Complex screens, Galerkin boundary element method, Quotient-space boundary element method, 22E46, 53C35, 57S20

## Abstract

A preconditioner is proposed for Laplace exterior boundary value problems on multi-screens. To achieve this, the quotient-space boundary element method and operator preconditioning are combined. For a fairly general subclass of multi-screens, it is shown that this approach paves the way for block diagonal Calderón preconditioners which achieve a spectral condition number that grows only logarithmically with decreasing mesh size, just as in the case of simple screens. Since the resulting scheme contains many more degrees of freedom than strictly required, strategies are presented to remove almost all redundancy without significant loss of effectiveness of the preconditioner. The performance of this method is verified by providing representative numerical results. Further numerical experiments suggest that these results can be extended to a much wider class of multi-screens that cover essentially all geometries encountered in practice, leading to a significantly reduced simulation cost.

## Introduction

**Fig. 1:**
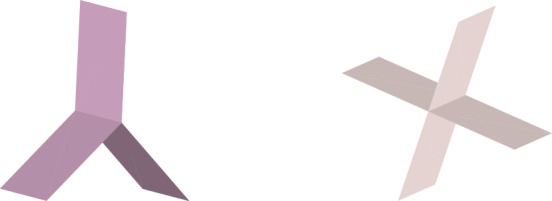
Two examples of multi-screen geometries

We are interested in the behaviour of potentials near multi-screens, which are geometries composed of essentially two-dimensional piecewise smooth surfaces joined together, as shown in Fig. 1. Hence, we consider the following Dirichlet and Neumann Laplace boundary value problems (BVPs) in the exterior of the multi-screen $\varGamma \subset \mathbb {R}^3$,

1 $$\begin{aligned} -\varDelta U= 0 \text { in } \mathbb {R}^3\setminus \overline{\varGamma }, \qquad U = g_D \quad \text { or } \quad \dfrac{\partial U}{\partial \textbf{n}} = f_N \quad \text { on } \varGamma , \end{aligned}$$

plus the decay condition $U(\textbf{x})= O(\Vert \textbf{x}^{-1} \Vert ) \text { as } \Vert \textbf{x}\Vert \rightarrow \infty ,$ where $\Vert \textbf{x}\Vert$ designates the Euclidean norm of a point $\textbf{x}$ in $\mathbb {R}^3$, $O(\cdot )$ the Landau symbol, and $g_D$ and $f_N$ are suitable boundary data.

Our goal is to solve these exterior BVPs efficiently by means of Galerkin boundary element methods (BEM) [29] and Calderón preconditioning [7, 31]. For this, we recast the BVPs as variational *first-kind* boundary integral equations (BIEs) for densities on the surface of the multi-screen.

For *simple screens* this approach is well established [29, Section 3.5.3]. Here, we call a simple screen an orientable, piecewise smooth two-dimensional manifold with boundary embedded in $\mathbb {R}^{3}$. For these geometries, the arising variational first-kind BIEs are known to be coercive [16, 17, 32] in Sobolev spaces of *jumps* of suitable field traces, in ${\widetilde{H}^{-\frac{1}{2}} (\varGamma )}$ and ${\widetilde{H}^{+\frac{1}{2}}(\varGamma )}$, respectively [27, Ch. 3]. For these trace spaces, conforming boundary element spaces are easily available, and they lead to Galerkin approximations and Calderón preconditioning whose numerical analysis is well-understood [23, 24, 28].

In contrast, the notion of jumps becomes problematic in multi-screens, since they are not globally orientable. For this reason, the tools from simple screens cannot be used straightforwardly on multi-screens. Many alternatives have been proposed to tackle this problem [5, 11–14, 35]. It is worth pointing out that at the time of writing, a rigorous analysis of these approaches in suitable trace spaces is not available. Furthermore, these approaches lead to ill-conditioned linear systems, yet are not amenable to preconditioning.

Fortunately, recent work by Claeys and Hiptmair offers the mathematical framework to overcome these difficulties [9]. The key idea is to see trace spaces from the perspective of quotient-spaces and to work with multi-valued traces. This new paradigm not only allows for a rigorous analysis, but it also paves the way for conforming Galerkin discretisation by means of quotient-space BEM, as proposed in [8]. Indeed, instead of trying to approximate jumps directly, the new approach relies on the Galerkin discretisation of multi-trace boundary element spaces. With this approach, the related BIEs give rise to Galerkin matrices with large null spaces comprised of single-trace functions. Since the right-hand-sides of the linear systems of equations are consistent, Krylov subspace iterative solvers like CG still converge to the right solution. We summarise these ideas and results in Sect. 2.

Now that the most fundamental issues have been solved, we are in the position to investigate how to improve the computational performance of quotient-space BEM for multi-screens. Indeed, one should note that the arising linear systems are ill-conditioned and that the number of conjugate gradient (CG) iteration counts increases with mesh refinement. Hence, a natural next step—and the main focus of this paper—is to devise preconditioners for multi-screen problems. In Sect. 4, we propose a simple preconditioning strategy based on opposite-order preconditioning, also known as Calderón preconditioning on closed surfaces. Moreover, we present the tools to understand the new preconditioner in the context of operator preconditioning. Numerical experiments confirm that this approach reduces considerably the number of CG iterations required to solve the system.

It is worth mentioning that an advantage of the quotient-space BEM approach is that minimal geometrical information is required. However, the disadvantage is that one pays with unnecessary computations due to the “doubling of degrees of freedom” underlying the discretisation of multi-valued traces. As an alternative, we dedicate Sect. 5 to discuss reduced quotient-space representations that require slightly more geometrical information, but lower computational effort while still rendering efficient Calderón preconditioning. Furthermore, we use the tools derived in Sect. 4 to provide some insight about the requirements that such reductions need to fulfil.

Last but not least, we should mention that another approach to preconditioning multi-screens has become available during the revision of this article [2]. We believe this confirms the problem at hand is relevant and that, as usual in mathematics, there are different ways of tackling a problem.

## Quotient-space perspective

We briefly summarise the new perspective introduced in [9, Section 4-6] and the quotient-space construction of boundary element (BE) spaces from [8]. Throughout this paper we focus on three dimensions, but it is worth mentioning that the method and analysis also carries over to 2*d*.

### Geometry

We begin by recalling the rigorous characterisation of *multi-screens* as given in [9, Sect. 2].

For this, the first concept we need to introduce is that of a Lipschitz (simple) screen in the sense of Buffa-Christiansen:

#### Definition 1

(Lipschitz Screen [9, Definition 2.1]) A *Lipschitz screen* is a subset $\varGamma \subset \mathbb {R}^3$ such that

- its closure $\overline{\varGamma }$ is a compact Lipschitz two-dimensional sub-manifold with boundary,
- Writing $\partial \varGamma$ for the the boundary of $\overline{\varGamma }$, we have that $\varGamma = \overline{\varGamma } {\setminus } \partial \varGamma$,
- there exists a finite covering $\mathcal {C}$ of $\overline{\varGamma }$ with cubes such that for each cube $C \in \mathcal {C}$, denoting by *a* the length of its sides, the following holds:
  - $\blacksquare$ If *C* contains a point in $\partial \varGamma$, there is an origin and an orthonormal basis of $\mathbb {R}^3$ in which the cube *C* can be identified with $(0, a)^3$ and there are uniformly Lipschitz continuous functions $\psi :\mathbb {R}\rightarrow \mathbb {R}$, and $\phi : \mathbb {R}^2 \rightarrow \mathbb {R}$, with values in (0, *a*) such that:
    2a $$\begin{aligned} \varGamma \cap C&= \lbrace (x, y, z) \in C \, : \, y < \psi (x), \, z = \phi (x, y)\rbrace , \end{aligned}$$
    2b $$\begin{aligned} \partial \varGamma \cap C&= \lbrace (x, y, z) \in C \, : \, y = \psi (x), \, z = \phi (x, y)\rbrace . \end{aligned}$$
  - $\blacksquare$ Otherwise, $\varGamma$ is the graph above $(0, a)^2$ of an uniformly Lipschitz continuous function $\phi : \mathbb {R}^2 \rightarrow \mathbb {R}$, with values in (0, *a*).

#### Definition 2

(Lipschitz Partition [9, Definition 2.2]) A *Lipschitz partition* of $\mathbb {R}^3$ is a finite collection of Lipschitz open sets $\left( \varOmega _j \right) _{j=0\ldots n}$ such that ${\mathbb {R}^3} = \cup _{j=0}^n \overline{\varOmega }_j$ and $\varOmega _j \cap \varOmega _k = \emptyset$, if $j \ne k$.

#### Definition 3

(Multi-screen [9, Definition 2.3]) A *multi-screen* is a subset $\varGamma \subset {\mathbb {R}^3}$ such that there exists a Lipschitz partition of ${\mathbb {R}^3}$ denoted $\left( \varOmega _j \right) _{j=0\ldots n}$ satisfying $\varGamma \subset \cup _{j=0}^n \partial {\varOmega _j}$ and such that for each $j = 0 \ldots n$, we have that the interior of $\overline{\varGamma }_j:= \overline{\varGamma } \cap \partial \varOmega _j$ is a Lipschitz screen.

From a numerical point of view, it will be convenient to classify multi-screens into three categories. For this, we first need to introduce the notion of irregular points on the boundary, as in [10].

#### Definition 4

(Irregular points [10, Sect. 6.1]) Let us consider $\partial \varGamma := {\overline{\varGamma }} {\setminus } \text {int}(\varGamma )$ and introduce the set of *regular points of the boundary* $\mathcal {P}_R( \partial \varGamma )$ defined as

$$\begin{aligned} \mathcal {P}_R(\partial \varGamma ) =&\lbrace x \in \partial \varGamma \text { such that } B_x \cap \varGamma = B_x \cap S \text { for some ball }B_x \nonumber \\  &\text { centred at } x \text { and some simple Lipschitz screen } S \rbrace . \end{aligned}$$

We define the set of *irregular points of the boundary* as

$$\begin{aligned} \mathcal {P}_I(\partial \varGamma )= \partial \varGamma \setminus \mathcal {P}_R(\partial \varGamma ). \end{aligned}$$

With this, we can classify our multi-screens as follows:

- **Type A:** $\varGamma$ is a multi-screen such that there exists an underlying Lipschitz partition $(\varOmega _j)_{j=0...n}$ of $\mathbb {R}^3$ with the property that $\partial \overline{\varGamma } _j \subseteq \partial \varGamma$ with $\varGamma _j$ as in Definition 3.
- **Type B:** $\varGamma$ is a multi-screen that has irregular points and that is not of type A.
- **Type C:** $\varGamma$ is a multi-screen without irregular points and that is not of type A.

**Fig. 2:**
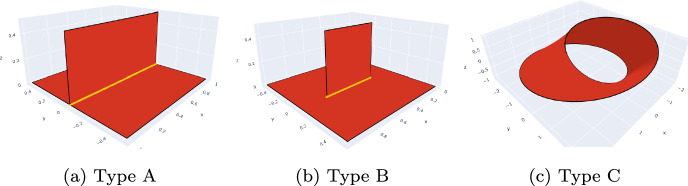
Multi-screens can be classified according to the location of their irregular points

Figure 2 provides examples of multi-screens in these three different classifications. In particular, Fig. 2c depicts a Möbius strip, which will not be discussed in this paper because its analysis is more cumbersome and it does not arise in applications. Indeed typical geometries that are approximated by multi-screens in applications are antennas, tail fins in aircrafts, and heat sinks. Since all of these correspond to multi-screens of Type A and Type B, we restrict ourselves to these two types.

### Trace spaces

For a multi-screen $\varGamma \subset {\mathbb {R}^3}$ we consider the following chains of nested Sobolev spaces^1^

3a $$\begin{aligned} H^1_{0,\varGamma }({\mathbb {R}^{3}})\subset H^1({\mathbb {R}^{3}}) \subset H^1({\mathbb {R}^{3}}\backslash \overline{\varGamma }), \end{aligned}$$

3b $$\begin{aligned} \textbf{H}_{0,\varGamma }(\textrm{div},{\mathbb {R}^{3}})\subset \textbf{H}(\textrm{div},{\mathbb {R}^{3}})\subset \textbf{H}(\textrm{div},{\mathbb {R}^{3}}\setminus \overline{\varGamma }), \end{aligned}$$

where the subscript $X_{0,\varGamma }$ indicates a space obtained as the closure in *X* of smooth functions/vectorfields compactly supported in ${ \mathbb {R}^{3}}\setminus \overline{\varGamma }$. All inclusions in (3) should be read as “is a closed subspace of”, which describe the associated quotient-spaces Hilbert spaces. With this, we can define the *multi-trace spaces* [9, Sect. 5]

4a $$\begin{aligned} \mathbb {H}^{+\frac{1}{2}}(\varGamma )&:= H^1({\mathbb {R}^3}\backslash \overline{\varGamma })/ H^1_{0,\varGamma }({\mathbb {R}^3}), \end{aligned}$$

4b $$\begin{aligned} \mathbb {H}^{- \frac{1}{2}}(\varGamma )&:= \textbf{H}(\textrm{div},{\mathbb {R}^3}\backslash \overline{\varGamma })/ \textbf{H}_{0,\varGamma }(\textrm{div},{\mathbb {R}^3}), \end{aligned}$$

and the *single-trace spaces* [9, Sect. 6.1]

5a $$\begin{aligned} H^{+\frac{1}{2}}([\varGamma ])&:= H^1({\mathbb {R}^3})/H^1_{0,\varGamma }({\mathbb {R}^3}), \end{aligned}$$

5b $$\begin{aligned} H^{-\frac{1}{2}}([\varGamma ])&:= \textbf{H}(\textrm{div},{\mathbb {R}^3})/ \textbf{H}_{0,\varGamma }(\textrm{div},{\mathbb {R}^3}). \end{aligned}$$

#### Remark 1

We note that $H^1({\mathbb {R}^3}\backslash \overline{\varGamma })$ and $\textbf{H}(\textrm{div},{\mathbb {R}^3}\setminus \overline{\varGamma })$ are spaces of functions attaining different values on both sides of $\varGamma$. This implies that functions in the multi-trace spaces $\mathbb {H}^{+\frac{1}{2}}(\varGamma )$ and $\mathbb {H}^{- \frac{1}{2}}(\varGamma )$ are multi-valued on $\varGamma$. In other words, they can take different values on both sides of $\varGamma$.

Since the spaces $H^{+\frac{1}{2}}([\varGamma ])$ and $H^{-\frac{1}{2}}([\varGamma ])$ are closed subspaces of $\mathbb {H}^{+\frac{1}{2}}(\varGamma )$ and $\mathbb {H}^{-\frac{1}{2}} (\varGamma )$, respectively [9, Proposition 6.2], we can also introduce the **jump spaces** [9, Sect. 6.2] as

6a $$\begin{aligned} \widetilde{H}^{+\frac{1}{2}}([\varGamma ])&:= \mathbb {H}^{+\frac{1}{2}}(\varGamma )/H^{+\frac{1}{2}}([\varGamma ]), \end{aligned}$$

6b $$\begin{aligned} \widetilde{H}^{-\frac{1}{2}}([\varGamma ])&:= \mathbb {H}^{- \frac{1}{2}}(\varGamma )/H^{-\frac{1}{2}}([\varGamma ]). \end{aligned}$$

#### Remark 2

It is worth mentioning that single-trace spaces are a generalisation of the spaces $H^{\pm \frac{1}{2}}(\varGamma )$ on simple screens. Indeed, definition (5) follows from the characterisation of trace spaces as the quotient between the domain of the Dirichlet and normal traces, and their kernels.

Then, multi-trace spaces are the counterpart of single-trace spaces when starting from the multi-valued spaces $H^1({\mathbb {R}^3}\backslash \overline{\varGamma })$ and $\textbf{H}(\textrm{div},{\mathbb {R}^3}{\setminus } \overline{\varGamma })$.

Finally, if one defines jump operators $[\cdot ]: \mathbb {H}^{\pm \frac{1}{2}} (\varGamma ) \rightarrow \widetilde{H}^{\pm \frac{1}{2}}([\varGamma ])$ as in [9, Def. 6.5], one gets that their kernels are the single-trace spaces. This motivates the characterisation of jump-spaces as the quotients (6).

Next, we consider the canonical surjections

7 $$\begin{aligned} \pi _D: H^1({\mathbb {R}^3}\backslash \overline{\varGamma }) \rightarrow \mathbb {H}^{+\frac{1}{2}}(\varGamma )\quad \textrm{and} \quad \pi _N: \textbf{H}(\textrm{div}, {\mathbb {R}^3}\backslash \overline{\varGamma }) \rightarrow \mathbb {H}^{- \frac{1}{2}}(\varGamma ), \end{aligned}$$

and, with $H^{1}(\varDelta ,{\mathbb {R}^3}{\setminus }\varGamma ) = \{ u \in H^{1}({\mathbb {R}^3}{\setminus }\varGamma ), \varDelta u \in L^2(\mathbb {R}^3) \}$, we define the relevant trace operators

$$\begin{aligned} \text {Dirichlet trace:}\quad  &   \gamma _{D}:H^{1}({\mathbb {R}^3} \setminus \varGamma )\rightarrow \mathbb {H}^{+\frac{1}{2}}(\varGamma ),  &   \gamma _{D}&:= \pi _{D} ,\\ \text {Neumann trace:}\quad  &   \gamma _{N}:H^{1}(\varDelta ,{\mathbb {R}^3} \setminus \varGamma )\rightarrow \mathbb {H}^{- \frac{1}{2}}(\varGamma ),  &   \gamma _{N}&:= \pi _{N}\circ {\textbf {grad}}. \end{aligned}$$

Moreover, we remark that they map onto $H^{+\frac{1}{2}}([\varGamma ])$ and $H^{-\frac{1}{2}}([\varGamma ])$ when restricted to ${H^1({\mathbb {R}^3})}$ and ${{H^1}({\varDelta }, {\mathbb {R}^3} )}$, respectively.^2^

As noted in [9, Sect. 5.1], Green’s Formula in $\mathbb {R}^3$ does not hold for elements of $H^1({\mathbb {R}^3}\setminus \overline{\varGamma })$ and $\textbf{H}(\textrm{div}, {\mathbb {R}^3}{\setminus }\overline{\varGamma })$. As these spaces underlie the definitions of $\mathbb {H}^{+\frac{1}{2}}(\varGamma )$ and $\mathbb {H}^{- \frac{1}{2}}(\varGamma )$, that implies we cannot use the usual $L^2$-duality pairing.

As a remedy, we introduce a bilinear pairing on $\mathbb {H}^{+\frac{1}{2}}(\varGamma )\times \mathbb {H}^{- \frac{1}{2}}(\varGamma )$:

8 $$\begin{aligned} \ll {u}, {p} \gg := \int \nolimits _{[\varGamma ]} {u}{p}\ d\sigma := \int _{\mathbb {R}^d \backslash \overline{\varGamma }} \textbf{p} \cdot \nabla u + u \textrm{div}(\textbf{p}) \ d\textbf{x}, \end{aligned}$$

with arbitrary representatives $u\in H^1({\mathbb {R}^3}{\setminus } \overline{\varGamma })$ and $\textbf{p} \in \textbf{H}(\textrm{div}, { \mathbb {R}^3}{\setminus }\overline{\varGamma })$ [9, Sect. 5.1].^3^ Note that this pairing induces the following *isometric dualities* [9, Prop. 5.1 and Sect. 6.2]

$$\begin{aligned} \mathbb {H}^{- \frac{1}{2}}(\varGamma )\cong \left( \mathbb {H}^{+\frac{1}{2}}(\varGamma )\right) ^\prime , \widetilde{H}^{-\frac{1}{2}}([\varGamma ])\cong \left( H^{+\frac{1}{2}}([\varGamma ])\right) ^\prime , \widetilde{H}^{+\frac{1}{2}}([\varGamma ])\cong \left( H^{-\frac{1}{2}}([\varGamma ])\right) ^\prime . \end{aligned}$$

Furthermore, the bilinear pairing (8) offers a characterisation of single-trace spaces through self-polarity:

#### Proposition 1

([9, Proposition 6.3]) For ${u} \in \mathbb {H}^{+\frac{1}{2}}(\varGamma )$ and ${p} \in \mathbb {H}^{ -\frac{1}{2}}(\varGamma )$ the following equivalences hold true:

$$\begin{aligned}&{u} \in H^{+\frac{1}{2}}([\varGamma ])\quad \Longleftrightarrow \quad {\ll {u}, {q} \gg } = 0 \quad \forall {q}\in H^{-\frac{1}{2}}([\varGamma ]),\\&{p} \in H^{-\frac{1}{2}}([\varGamma ])\quad \Longleftrightarrow \quad {\ll {v}, {p} \gg } = 0 \quad \forall {v}\in H^{+\frac{1}{2}}([\varGamma ]). \end{aligned}$$

#### Remark 3

These polarity properties may seem surprising starting from the quotient space definition of the multi-trace spaces. However, thinking of the interpretation of the pairing (8) as an $L^2$-type pairing on an inflated multi-screen, they make sense: $u \in H^{+\frac{1}{2}}([\varGamma ])$ is *even* when crossing $\varGamma$ and $p \in H^{-\frac{1}{2}}([\varGamma ])$ is *odd* when crossing $\varGamma$ (because the normal changes direction). The result is that contributions from opposite sides of $\varGamma$ cancel.

### Weakly singular and hypersingular BIEs

Let ${\mathcal {G}(\textbf{z}):= \dfrac{1}{4 \pi \Vert \textbf{z}\Vert }}$ be the fundamental solution of the Laplace equation in $\mathbb {R}^{3}$. For $\textbf{x} \notin \varGamma$, let $\mathcal {G}_{\textbf{x}}(\textbf{y}):= \chi _{\textbf{x}}(\textbf{y}) \mathcal {G}(\textbf{x}-\textbf{y})$ with $\chi _{\textbf{x}}: \mathbb {R}^3 \rightarrow \mathbb {R}$ a smooth cut-off function that is 1 in a neighborhood of $\varGamma$ and 0 in a neighborhood of $\textbf{x}$ as in [9, Sect. 8]. This allows the definition of the single and double layer potentials by

9 $$\begin{aligned} {\text {SL}}\phi (x) := \ll \gamma _{D} \mathcal {G}_x, \phi \gg , \quad {\text {DL}}v(x) := -\ll \gamma _{N} \mathcal {G}_x, v \gg . \end{aligned}$$

The weakly singular and hypersingular boundary integral operator (BIO) are the continuous operators $\textsf{V}_{0}:= \gamma _D \circ {\text {SL}}: \mathbb {H}^{- \frac{1}{2}}(\varGamma )\rightarrow \mathbb {H}^{+\frac{1}{2}}(\varGamma ), \quad \textsf{W}_{0}:= \gamma _N \circ {\text {DL}}: \mathbb {H}^{+\frac{1}{2}}(\varGamma )\rightarrow \mathbb {H}^{- \frac{1}{2}}(\varGamma ).$ For sufficiently smooth arguments, the corresponding bilinear forms admit weakly singular representations given by [8, Sect. 3]

10 $$\begin{aligned} \ll \textsf{V}_{0} \phi , \psi \gg =&\int _{[\varGamma ]} \int _{[\varGamma ]} \mathcal {G}(\textbf{y}-\textbf{x}) \phi (y) \psi (x) d\sigma (\textbf{y})d\sigma (\textbf{x}), \end{aligned}$$

11 $$\begin{aligned} \ll \textsf{W}_{0} v, p \gg =&\int _{[\varGamma ]} \int _{[\varGamma ]} \mathcal {G}(\textbf{y}-\textbf{x}) {\mathrm {\textbf{curl}}_\varGamma \,{{v}}(\textbf{y})\cdot \mathrm {\textbf{curl}}_\varGamma \,{{p}}(\textbf{x})} d\sigma (\textbf{y})d\sigma (\textbf{x}), \end{aligned}$$

In order to solve the Dirichlet Laplace BVP, we solve the following variational BIE: Given ${g}_D \in H^{+\frac{1}{2}}([\varGamma ])$, find ${{\phi }} \in \mathbb {H}^{- \frac{1}{2}}(\varGamma )$ such that

12 $$\begin{aligned} \ll {\textsf{V}_{0}} {{\phi }}, {{\psi }} \gg = \ll {{g}_D}, {{\psi }} \gg \quad \forall {{\psi }} \in \mathbb {H}^{- \frac{1}{2}}(\varGamma ). \end{aligned}$$

To solve the Neumann Laplace BVP, we solve the variational BIE: Given ${f}_N\in H^{-\frac{1}{2}}([\varGamma ])$, find ${u} \in \mathbb {H}^{+\frac{1}{2}}(\varGamma )$ such that

13 $$\begin{aligned} \ll {\textsf{W}_{0}} {{v}}, {{p}} \gg = \ll {{f}_N}, {{p}} \gg \quad \forall {{p}} \in \mathbb {H}^{+\frac{1}{2}}(\varGamma ). \end{aligned}$$

We conclude this section by recalling some properties of these BIEs: First, as a consequence of the polarity from Proposition 1, we get that

#### Lemma 1

([8, Lemma 3.2]) The nullspaces of ${\textsf{V}_{0}}$ and ${\textsf{W}_{0}}$ agree with ${H^{-\frac{1}{2}}([\varGamma ])}$ and $H^{+\frac{1}{2}}([\varGamma ])$, respectively.

In analogy to the situation on simple screens, we have

#### Proposition 2

The operators $\textsf{V}_0:\widetilde{H}^{-\frac{1}{2}}([\varGamma ])\rightarrow H^{+\frac{1}{2}}([\varGamma ])$ and $\textsf{W}_0:\widetilde{H}^{+\frac{1}{2}}([\varGamma ])\rightarrow H^{-\frac{1}{2}}([\varGamma ])$ are elliptic, i.e.

14 $$\begin{aligned} {\ll \textsf{V}_{0}q,{{q}} \gg }&\ge {\alpha }_{\textsf{V}} \Vert {q} \Vert ^2_{\widetilde{H}^{-\frac{1}{2}}([\varGamma ])} \quad \forall {q} \in \widetilde{H}^{-\frac{1}{2}}([\varGamma ]), \end{aligned}$$

15 $$\begin{aligned} {\ll \textsf{W}_{0}v,v \gg }&\ge {\alpha }_{\textsf{W}} \Vert {v} \Vert ^2_{\widetilde{H}^{+\frac{1}{2}}([\varGamma ])} \quad \forall {v} \in \widetilde{H}^{+\frac{1}{2}}([\varGamma ]), \end{aligned}$$

with ${\alpha }_{\textsf{V}}, {\alpha }_{\textsf{W}}>0$ depending only on $\varGamma$.

#### Proof

The proof is similar to that of [9, Prop. 8.7] but setting $\psi$ to be the single layer potential for the Laplacian and using the fact that $\varDelta \psi = 0$ and that $\vert \psi \vert _{H^1(\mathbb {R}^3{\setminus } \overline{\varGamma })}$ is equivalent to $\Vert \psi \Vert _{H^1(\varDelta ,\mathbb {R}^3 \setminus \overline{\varGamma })}$ [29, Thm. 2.10.10]. $\square$

The previous results combined with continuity of the operators give us

#### Proposition 3

([9, Prop. 8.9]) The operators $\textsf{V}_0:\widetilde{H}^{-\frac{1}{2}}([\varGamma ])\rightarrow H^{+\frac{1}{2}}([\varGamma ])$ and $\textsf{W}_0:\widetilde{H}^{+\frac{1}{2}}([\varGamma ])\rightarrow H^{-\frac{1}{2}}([\varGamma ])$ are isomorphisms.

Additionally, these operators remain well-defined on the multi-trace spaces $\mathbb {H}^{- \frac{1}{2}}(\varGamma )$ and $\mathbb {H}^{+\frac{1}{2}}(\varGamma )$, respectively. However, Lemma 1 implies that they have non-trivial nullspaces when considered on multi-trace spaces. Although this excludes uniqueness of solutions for (12) and (13), Proposition 1 still provides existence, since ${g}_{D}\in H^{+\frac{1}{2}}([\varGamma ])$ and ${f}_{N}\in H^{-\frac{1}{2}}([\varGamma ])$ guarantees consistency of the right-hand side linear forms: they vanish on the single-trace spaces.

## Operator preconditioning on quotient-space BEM

In order to explain what changes in the quotient-space BEM setting, we recall the essential ingredients of operator preconditioning as presented in [22]: Let $\mathbb {X}$, $\mathbb {Y}$ be Banach spaces, and consider the finite-dimensional subspaces $\mathbb {X}_h \subset \mathbb {X}$ and $\mathbb {Y}_h \subset \mathbb {Y}$ with dimensions $N:=\dim \mathbb {X}_h$ and $M:=\dim \mathbb {Y}_h$, and bases $(\varphi _{i})_{i=1}^N$ and $(\phi _{i})_{i=1}^M$, respectively. Further, let $\textsf{a}\in L(\mathbb {X}\times \mathbb {X}, {\mathbb {R}})$, $\textsf{b}\in L(\mathbb {Y}\times \mathbb {Y}, {\mathbb {R}})$ and $\textsf{m}\in L(\mathbb {X}\times \mathbb {Y}, {\mathbb {R}})$ be continuous bilinear forms satisfying discrete inf-sup conditions:

16 $$\begin{aligned} \sup _{v_h\in X_h} \dfrac{\vert \textsf{a}(u_h, v_h) \vert }{\Vert v_h \Vert _\mathbb {X}}&\ge \alpha _A \Vert u_h \Vert _{\mathbb {X}}, \quad \forall u_h \in \mathbb {X}_h,\end{aligned}$$

17 $$\begin{aligned} \sup _{w_h\in Y_h} \dfrac{\vert \textsf{b}(q_h, w_h) \vert }{\Vert w_h \Vert _\mathbb {Y}}&\ge \alpha _B \Vert q_h \Vert _\mathbb {Y}, \quad \forall q_h \in \mathbb {Y}_h,\end{aligned}$$

18 $$\begin{aligned} \sup _{w_h\in Y_h} \dfrac{\vert \textsf{m}(v_h, w_h) \vert }{\Vert w_h \Vert _\mathbb {Y}}&\ge \alpha _M \Vert v_h \Vert _{\mathbb {X}}, \quad \forall v_h \in \mathbb {X}_h. \end{aligned}$$

If $N=M$, then [22, Theorem 2.1] implies that the associated Galerkin matrices $\textbf{A}_{h}:= \left( \textsf{a}(\varphi _i, \varphi _j ) \right) _{i,j=1}^{N}, \; \textbf{B}_{h}:= \left( \textsf{b}(\phi _i, \phi _j ) \right) _{i,j=1}^{N}, \; \textbf{M}_{h}:= \left( \textsf{m}(\varphi _i, \phi _j) \right) _{i,j=1}^{N},$ satisfy

19 $$\begin{aligned} \kappa _{sp} (\textbf{M}_{h}^{-1}\textbf{B}_{h}\textbf{M}_{h}^{-{T}}\textbf{A}_{h}) \le \frac{\Vert \textsf{a}\Vert \Vert \textsf{b}\Vert \Vert \textsf{m}\Vert ^2}{{\alpha _A} {\alpha _B} {\alpha _M}^2}, \end{aligned}$$

where $\kappa _{sp}$ designates the spectral condition number and $\Vert \cdot \Vert$ denotes the corresponding operator norms.

From (19), the idea is that if we are solving (12), the Galerkin matrix of $\textsf{V}_0$ would play the role of $\textbf{A}_h$ and we would need to find a suitable bilinear form $\textsf{b}$ such that the spectral condition number is as small as possible if we precondition the resulting linear system with $\textbf{P}_h = \textbf{M}_{h}^{-1}\textbf{B}_{h}\textbf{M}_{h}^{-{T}}$. Analogously, if we are solving (13), the Galerkin matrix of $\textsf{W}_0$ would play the role of $\textbf{A}_h$ and we would need a different choice of $\textsf{b}$.

Hence, the first question is: which Galerkin matrix one should consider? In other words, what discretisation should we choose? The answer following quotient-space BEM is to discretise the multi-trace spaces. However, due to Lemma 1, the bilinear forms of $\textsf{V}_0$ and $\textsf{W}_0$ will not satisfy its inf-sup condition on their discrete multi-trace space. We therefore have to extend (19) to use operator preconditioning for (12) and (13).

As usual, bounding the spectral condition number entails bounding the largest eigenvalue $\lambda _{\max }:= \lambda _{\max }(\textbf{M}_h^{-1}\textbf{B}_{h}\textbf{M}_h^{-T}\textbf{A}_{h})$ from above and the smallest $\lambda _{\min }:= \lambda _{\min }(\textbf{M}_h^{-1}\textbf{B}_{h} \textbf{M}_h^{-T}\textbf{A}_{h})$ from below. However, when using quotient-space BEM, we have to consider the spectral condition number away from the kernel of $\textbf{A}_{h}$, i.e., $\tilde{\kappa }_{sp}(\textbf{M}_h^{-1}\textbf{B}_{h}\textbf{M}_h^{-T}\textbf{A}_{h}) = \lambda _{\max }/ \widetilde{\lambda _{\min }}$, where $\widetilde{\lambda _{\min }}$ is the smallest **non-zero** eigenvalue of $\textbf{M}_h^{-1}\textbf{B}_{h}\textbf{M}_h^{-T}\textbf{A}_{h}$, since this quantity determines the convergence of CG on singular systems [20, 26]. In order to write these bounds, we need to introduce some notation first.

Let $\textsf{A}_h :\, \mathbb {X}_h\rightarrow \mathbb {X}_h^\prime$, $\textsf{B}_h \,:\, \mathbb {Y}_h\rightarrow \mathbb {Y}_h^\prime$ and $\textsf{M}_h \,:\, \mathbb {X}_h\rightarrow \mathbb {Y}_h^\prime$ be the bounded linear operators associated to the bilinear forms $\textsf{a}$, $\textsf{b}$ and $\textsf{m}$, respectively.

For $\lambda _{\max }$ we proceed in the classical way and arrive to

20 $$\begin{aligned} \lambda _{\max }&\le \Vert \textsf{M}_h^{-1} \Vert ^2\Vert \textsf{B}_h \Vert \Vert \textsf{A}_h \Vert = \alpha _{{M}}^{{-}2} \Vert \textsf{B}_h \Vert \Vert \textsf{A}_h \Vert . \end{aligned}$$

For ${\widetilde{\lambda }}_{\min }$ we have to take a slightly different approach since we need to restrict $\textsf{A}_h$ to the space where its corresponding bilinear form $\textsf{a}$ satisfies a discrete inf-sup condition. Moreover, we have to establish when the discrete inf-sup condition will bound the smallest eigenvalue. We study this in the next Lemma.

### Lemma 2

(Discrete inf-sup constant in the quotient space norm) Let $\mathbb {X}$ be a Hilbert space. Let $\textsf{a}$ be a continuous bilinear form on $\mathbb {X}\times \mathbb {X}$. Let $X\subseteq \mathbb {X}$ be both the left and right nullspace of $\textsf{a}$. Let $\mathbb {X}_h$ be a finite dimensional subspace of $\mathbb {X}$ and $\textsf{A}_h \,:\, \mathbb {X}_h\rightarrow \mathbb {X}_h^\prime$ the bounded linear operator associated to $\textsf{a}$. We assume that ${X_h}$ is nullspace conforming to $\textsf{a}$ in the sense that $X_h:= \ker \textsf{A}_h = \ker \textsf{A}'_h$ is a linear subspace of $X\cap \mathbb {X}_h\subseteq X$.

If $\textsf{a}$ satisfies a discrete inf-sup condition in ${\mathbb {X}_h/X \times \mathbb {X}_h/X}$ with constant $\alpha _{\textsf{a}} > 0$ and if the norms on $\mathbb {X}/X$ and $\mathbb {X}_h/X_h$ are equivalent, i.e. there exists $c_{eq}> 0$, such that for all $u_h \in \mathbb {X}_h$

21 $$\begin{aligned} \Vert u_h \Vert _{\mathbb {X}/X} \le \Vert u_h \Vert _{\mathbb {X}/X_h} \le c_{eq} \Vert u_h \Vert _{\mathbb {X}/X}, \end{aligned}$$

then

22 $$\begin{aligned} \sup _{v_h \in \mathbb {X}_h\setminus \{0\}} \frac{\textsf{a}(u_h,v_h)}{ \Vert v_h\Vert _{\mathbb {X}}} \ge {\frac{{\alpha _{\textsf{a}}}}{c_{eq}}} \Vert u_h\Vert _{\mathbb {X}/X}. \end{aligned}$$

### Proof

We have, with $X_h^\perp = \{ u_h \in \mathbb {X}_h, \forall v_h \in X_h, (u_h, v_h)_\mathbb {X}= 0 \}$,

23 $$\begin{aligned} \sup _{v_h \in \mathbb {X}_h\setminus \{0\}} \frac{\textsf{a}(u_h,v_h)}{ \Vert v_h\Vert _{\mathbb {X}}} \ge \sup _{\tilde{v}_h \in X_h^\perp \setminus \{0\}} \frac{\textsf{a}(u_h,\tilde{v}_h)}{ \Vert \tilde{v}_h\Vert _{\mathbb {X}}}. \end{aligned}$$

For $\tilde{v}_h \in X_h^\perp$, we have $\Vert \tilde{v}_h\Vert _{\mathbb {X}} = \Vert \tilde{v}_h\Vert _{\mathbb {X}/X_h}$ (e.g. [6, Eq. (4)]), and so

24 $$\begin{aligned} \sup _{\tilde{v}_h \in X_h^\perp \setminus \{0\}} \frac{\textsf{a}(u_h,\tilde{v}_h)}{\Vert \tilde{v}_h\Vert _{\mathbb {X}}}&=\sup _{\tilde{v}_h \in \mathbb {X}_h/X_h\setminus \{0\}} \frac{\textsf{a}(u_h,\tilde{v}_h)}{ \Vert \tilde{v}_h\Vert _{\mathbb {X}/X_h}}, \end{aligned}$$

where we have identified $X_h^\perp$ and $\mathbb {X}_h/X_h$. Finally, by the norm equivalence (21) and the discrete inf-sup condition, we have that

25 $$\begin{aligned} \sup _{v_h \in \mathbb {X}_h\setminus \{0\}} \frac{\textsf{a}(u_h,v_h)}{ \Vert v_h \Vert _{\mathbb {X}}}&\ge c_{eq}^{-1} \, \sup _{\tilde{v}_h \in \mathbb {X}_h/X_h\setminus \{0\}} \frac{\textsf{a}(u_h, \tilde{v}_h)}{ \Vert \tilde{v}_h\Vert _{\mathbb {X}/X}}&\ge \frac{\alpha _{\textsf{a}}}{c_{eq}} \Vert u_h \Vert _{\mathbb {X}/X}. \end{aligned}$$

$$\square $$

Note that inequality (21) holds in our application by virtue of Lemma 7.

Using Lemma 2 and the properties of the related bilinear forms, we can bound ${\widetilde{\lambda }}_{\min }$ as follows

26 $$\begin{aligned} {\widetilde{\lambda }}_{\min }&{\ge } \inf _{u_h\in \mathbb {X}_h/X_h\setminus \{0\}} \dfrac{\Vert \textsf{M}_h^{-1}\textsf{B}_{h}\textsf{M}^{-*}_h{\textsf{A}_h} u_h\Vert _{ \mathbb {X}}}{\Vert u_h \Vert _{\mathbb {X}}} \ge \alpha _{\textsf{M}^{-1}}^2 \alpha _{\textsf{B}} {\frac{\alpha _{A}}{c_{eq}}}{\ge \frac{\alpha _{\textsf{B}}\alpha _{A}}{\Vert \textsf{M}\Vert ^2 c_{eq}}}. \end{aligned}$$

Finally, we combine (20) and (26) and get

27 $$\begin{aligned} {\tilde{\kappa }}_{sp}(\textbf{M}^{-1} \textbf{B}_{h}\textbf{M}^{-T} \textbf{A}_{h}) \le \dfrac{{c_{eq}} \Vert \textsf{B}\Vert \Vert \textsf{A}\Vert {\Vert \textsf{M}\Vert ^2}}{ \alpha _{\textsf{B}} {\alpha _{A}}{\alpha _{\textsf{M}}^2}}. \end{aligned}$$

### Remark 4

For the discrete quotient norm to be bounded from below by the continuous quotient norm, it suffices that $X_h \subseteq X \cap \mathbb {X}_h$; equality is not required. This opens the door to reduction schemes for the multi-trace space variational formulation. A reduction scheme is a choice $\mathbb {X}_h^\circ \subset \mathbb {X}_h \subset \mathbb {X}$ with corresponding discrete left/right nullspace $X_h^\circ$ such that (i) $X_h^\circ \subset X$ and (ii) $\mathbb {X}_h^\circ / X_h^\circ = \mathbb {X}_h / X_h$. Such a choice leads, on the one hand, to approximations in the jump space of equal quality, and, on the other hand, does not preclude the construction of efficient operator preconditioners. We will discuss this again in Sect. 5.

## Calderón preconditioning for multi-screens

As already mentioned in the introduction, the linear systems arising from the discretisation of (12) and (13) using Quotient-space BEM are ill-conditioned, which causes that the number of CG iteration counts increases with mesh refinement. One should note that this is not a particularity of Quotient-space BEM. Indeed, we usually encounter this difficulty when using low-order BEM discretisation of first-kind integral equations on simple screens and closed surfaces. In those cases, one typically remedies the problem by using so-called Calderón preconditioning, which combines Calderón identities with operator preconditioning to build a convenient and effective preconditioner [7, 22, 31].

In this paper, we will extend this approach and devise Calderón preconditioners for the problem at hand. For the sake of presentation, we will discuss the details for the case of the Hypersingular operator $\textsf{W}_0$ and note that one can proceed analogously to precondition the weakly singular operator $\textsf{V}_0$.

### Discretisation

When considering the Neumann problem (13), we want to find solutions *v* in $\widetilde{H}^{+\frac{1}{2}}([\varGamma ])$. The idea of Quotient-space BEM is to discretise the multi-trace space $\mathbb {H}^{+\frac{1}{2}}(\varGamma )$ instead.

**Fig. 3:**
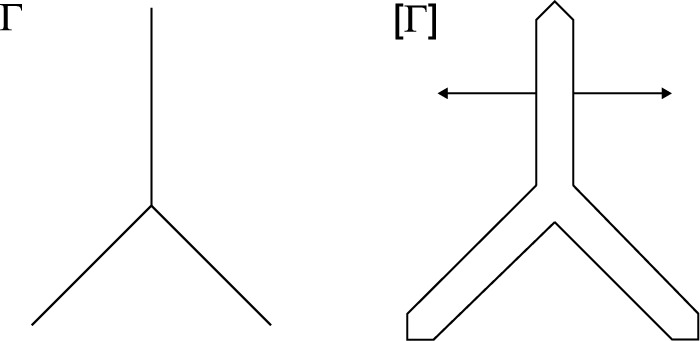
“Inflating” a 2D multi-screen

As mentioned in Remark 1, multi-trace spaces can take different values on both sides of $\varGamma$. One way to grasp this is to imagine an “infinitesimally inflated” screen, as illustrated in Fig. 3 for a 2D multi-screen. With this, one can intuitively understand the trace spaces introduced in Sect. 2.2 as follows:

- $\mathbb {H}^{+\frac{1}{2}}(\varGamma )$ can be seen as a standard Dirichlet trace space on the surface of the “inflated screen”. Similarly, $\mathbb {H}^{- \frac{1}{2}}(\varGamma )$ can be viewed as the standard Neumann trace space on the surface of the “inflated screen”.
- The single-trace space $H^{+\frac{1}{2}}([\varGamma ])$ simply consists of single-valued functions on $\varGamma$. One can follow the same intuition for $H^{-\frac{1}{2}}([\varGamma ])$, however, its right interpretation as a single-valued normal component requires that one fixes a local normal $\textbf{n}$ on $\varGamma$.

It is worth noting that this depiction via the “inflated screen” is only meant to help the reader to visualise the related spaces. Furthermore, every time we resort to this intuition, the thickness of the “inflated screen” is indeed zero.

**Fig. 4:**
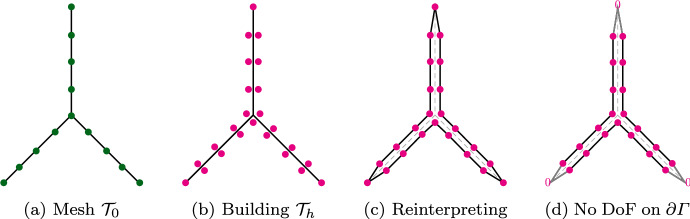
Illustration of virtual mesh for 2*D* multi-screen (please note that here the thickness of the virtual meshes is non-zero only to simplify the picture.)

Following this intuition, a simple way to implement the multi-trace space in an already existing BEM code, is via a *triangular virtual surface mesh of* $\varGamma$, as defined in [8, Sect. 4.1]. In essence, this virtual mesh is built from a triangulation $\mathcal {T}_0$ of $\varGamma$. Then, for every triangle $K \in \mathcal {T}_0$ one creates two copies $K^+$ and $K^-$ with the same geometry but to be regarded as different entities. The reader may imagine $K^+$ and $K^-$ as the two faces of *K*. In addition, one defines $\textbf{n}_K$ as the unit normal vector of *K*, and labels the copies such that $\textbf{n}_K$ points from $K^-$ to $K^+$. In this way, $K^+$ is endowed with $\textbf{n}_K$ and $-\textbf{n}_K$ is assigned to $K^-$. Finally, the union of these oriented triangles forms the set underlying what we call the virtual surface mesh of $\varGamma$. Figure 4 illustrates this idea for a 2D multi-screen (where triangles are replaced by segments of the mesh). We refer to [8, Sect. 4.1] for further details.

Let $\mathcal {T}_h$ be a triangular virtual surface mesh of $\varGamma$, with target element size *h*, and let $\check{\mathcal {T}}_h$ be its dual as realised on the barycentric refinement [4]. The BE spaces above could be chosen as [30, Sect. 2.2]

- $\mathbb {X}_h(\varGamma )= \mathcal {S}^{1,0}(\mathcal {T}_h)$: piecewise linear “continuous” functions on $\mathcal {T}_h$,
- $\mathbb {Y}_h(\varGamma )= \mathcal {S}^{0,-1}(\check{\mathcal {T}}_h)$: piecewise constant functions on $\check{\mathcal {T}}_h$,

yet we will need a different choice for our preconditioner to work, as we will discuss in the next section.

### Implementation

Note that by construction of the virtual mesh, which can be understood as a triangulation of a closed surface (see Fig. 4c), the space $\mathcal {S}^{1,0}(\mathcal {T}_h)$ has one degree of freedom at the vertices in $\mathcal {T}_h \cap \partial \varGamma$. Since solutions for the hypersingular equation (13) belong to $\widetilde{H}^{+\frac{1}{2}}([\varGamma ])$, we know they will be zero on $\partial \varGamma$ [8]. Indeed, because functions in $\widetilde{H}^{+\frac{1}{2}}([\varGamma ])$ are only determined up to contributions in $H^{+\frac{1}{2}}([\varGamma ])$, degrees of freedom on $\partial \varGamma$ (which by construction are in $H^{+\frac{1}{2}}([\varGamma ])$) can be safely deleted. Hence, instead of working with $\mathcal {S }^{1,0}(\mathcal {T}_h)$, we consider $\mathcal {S}_{0}^{1,0}(\mathcal {T}_h)\subset \mathbb {H}^{+\frac{1}{2}}(\varGamma )$: piecewise linear “continuous” functions on the inflated screen that are zero on $\partial \varGamma$, as shown in Fig. 4d.

**Fig. 5:**
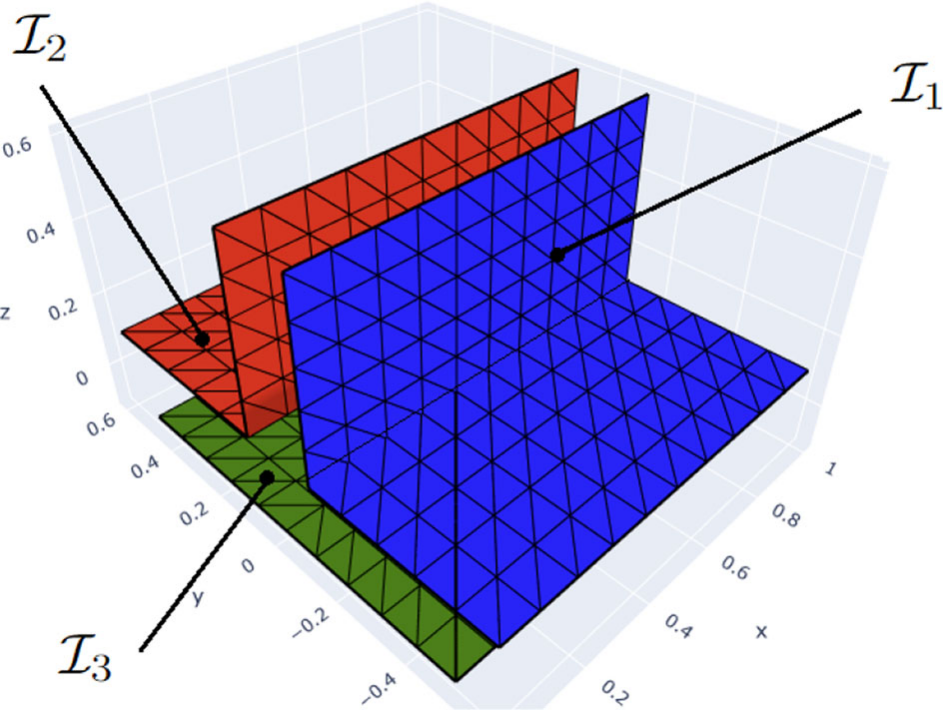
Back-front (decoupled) conforming mesh on the multi-screen

When dealing with multi-screens of **type A**, this will have the computational advantage of allowing us to decouple the BE spaces on each side of the triangular virtual surface mesh $\mathcal {T}_h$, as depicted in Figs. 4d and 5.

Let us illustrate how we implemented these BE spaces on a multi-screen $\varGamma$ that is the union of three simple screens ${\mathcal {M}_i}, i=1,2,3$ meeting at a junction:

1. We define the inflated multi-screen $[\varGamma ]$ as
  28 $$\begin{aligned} [\varGamma ] = \cup _{l=1}^{3} \mathcal {I}_{l} \end{aligned}$$
  with $\mathcal {I}_l = {\mathcal {M}_{l} \cup \mathcal {M}_{l+1}}$. The normal on $\mathcal {I}_l$ is chosen *outward*. Each simple screen ${\mathcal {M}_i}$ appears once as the *front* and once as the *back* of the multi-screen (see Fig. 5).
2. For $i=1,2,3$, we create the triangular surface mesh ${\mathcal {M}_{i,h}}$ of ${\mathcal {M}_{i}}$ with target element size *h*, and such that the meshes ${\mathcal {M}_{i,h}}$ for $i=1,2,3$ match up along the junction. The simple screens $\mathcal {I}_l$ inherit this mesh. In other words, we have ${\mathcal {I}_{l,h}= \mathcal {M}_{l,h} \bigcup \mathcal {M}_{l+1,h}, \, l=1,2,3}$.
3. The discrete *primal* multi-trace space $\mathbb {X}_h(\varGamma )$ is built as the product of these spaces, i.e.
  29 $$\begin{aligned} \mathbb {X}_h(\varGamma )= \mathcal {S}_{0}^{1,0}(\mathcal {T}_h) = {\bigotimes }_{l=1}^{3} \mathcal {S}_{0}^{1,0}(\mathcal {I}_{l,h}). \end{aligned}$$
4. Construct the dual BE spaces on the simple screens $\mathcal {I}_l$ following the cue from [4]. For this, let $\check{\mathcal {I}}_{l,h}$ denote the dual barycentric mesh to $\mathcal {I}_{l,h}$, built as in [25, Definition 2]. Then we introduce the space $\mathcal {S}^{0,-1}(\check{\mathcal {I}}_{l,h})\subset \mathbb {H}^{-\frac{1}{2}}(\mathcal {I}_l)$ of piecewise constant functions supported by the dual cells of $\check{\mathcal {I}}_{l,h}$ that correspond to nodes not on the boundary of $\mathcal {I}_l$. In particular we have that $\dim \mathcal {S}^{0,-1}(\check{\mathcal {I}}_{l,h}) = \dim \mathcal {S}^{1,0}_0(\mathcal {I}_{l,h})$.
5. The discrete *dual* multi-trace space $\mathbb {Y}_h(\varGamma )$ is built as the product of these dual spaces, i.e.
  30 $$\begin{aligned} \mathbb {Y}_h(\varGamma )= \mathcal {S}^{0,-1}(\check{\mathcal {T}}_h)= {\bigotimes }_{l=1}^{3} \mathcal {S}^{0,-1}(\check{\mathcal {I}}_{l,h}). \end{aligned}$$
  By construction, we have that $N:= \dim (\mathbb {Y}_h(\varGamma )) =\dim (\mathbb {X}_h(\varGamma ))$.

#### Remark 5

The description of discrete multi-trace spaces (29) and (30) is not valid for multi-screens of Types B and C.

### Block diagonal Calderón preconditioner

Based on Calderón preconditioning for closed surfaces and its applicability to simple screens, one could think of preconditioning the hypersingular operator $\textsf{W}_0$ with the weakly singular operator $\textsf{V}_0$. However, it is clear from Lemma 1 that $\textsf{V}_0$ will not do the job.

As an alternative, we propose to use Calderón preconditioning blockwise, under the considerations of the previous sections. Let $\mathbb {X}_h(\varGamma )= {\text {span}}\lbrace \varphi _k \rbrace _{{k=1}}^{N}$ and consider the Galerkin matrix for the hypersingular operator, i.e.,

31 $$\begin{aligned} \textbf{W}_{h}[i,j]=\ll \textsf{W}_{0} \varphi _j, \varphi _i\gg , \quad i,j =1,\dots ,N. \end{aligned}$$

Then, we will build a preconditioner for $\textbf{W}_{h}$ based on the block matrix

32 $$\begin{aligned} \textbf{B}_{h}^{\textsf{V}} := \left( \begin{array}{c c c} \check{\textbf{V}}_{h,1} &  \textbf{0} &  \textbf{0} \\ \textbf{0} & \check{\textbf{V}}_{h,2} &  \textbf{0} \\ \textbf{0} & \textbf{0} & \check{\textbf{V}}_{h,3}\\ \end{array}\right) , \end{aligned}$$

where $\check{\textbf{V}}_{h,l}[i,j]=\langle \textsf{V}_{0} \check{\psi }_j, \check{\psi }_i\rangle _{\mathcal {I}_{l}}$ with $\check{\psi }_i,\check{\psi }_j$ in the standard basis of $\mathcal {S}^{0,-1}(\check{\mathcal {I}}_{l,h})$ for $l=1,2,3$, and $\langle \cdot , \cdot \rangle _{\mathcal {I}_{l}}$ denotes the usual $L^2$-duality pairing over $\mathcal {I}_{l}$.

The motivation to consider this $\textbf{B}_{h}^{\textsf{V}}$ is that the choice of discrete spaces from (29) and (30) allows us to decouple what is happening on the dual space of each simple screen $\mathcal {I}_{l}$. Furthermore, they would agree with the standard discretisation of the jump spaces on simple screens. More concretely, we have that $\mathcal {S}_{0}^{1,0}({\mathcal {I}}_{l,h})\subset \widetilde{H}^{\frac{1}{2}}(\mathcal {I}_{ l})$ and $\mathcal {S}^{0,-1}(\check{\mathcal {I}}_{l,h}) \subset \widetilde{H}^{-\frac{1}{2}} (\mathcal {I}_{l})$.

In order to analyse the impact of this preconditioner in the number of PCG iteration counts, we aim to bound the resulting condition number. For this, we first need to make some assumptions.

**Fig. 6:**
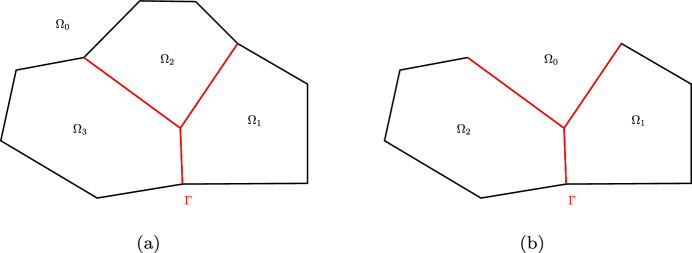
Example of two Lipschitz partitions for $\varGamma$ being a 2*D* multi-screen with a triple junction

Let us begin by noticing that the Lipschitz partition $\left( \varOmega _j\right) _{ j=0\ldots n}$ such that $\varGamma \subset \cup _{j=0}^m \partial \varOmega _j$ is not unique. We illustrate this for a two-dimensional multi-screen $\varGamma$ with a triple junction in Fig. 6. Nevertheless, for the multi-screens considered in this paper, one can always find a Lipschitz partition such that the Lebesgue measure ${\text {meas}}(\varGamma \cap \partial \varOmega _i)>0$ for all $i=0,\dots ,m$. In other words, we can always assume we have the configuration corresponding to Fig. 6b. For simplicity of the proofs, this is the type of Lipschitz partitions that we will consider. This and the particular order of the domains is stated in the following:

#### Assumption 1

Let $\left( \varOmega _j \right) _{j=0\ldots n}$ be a Lipschitz partition in $\mathbb {R}^3$. We assume $\varGamma$ to be a multi-screen such that $\varGamma \subset \cup _{j=0}^m\partial \varOmega _j$ and ${\text {meas}}(\varGamma \cap \partial \varOmega _i)>0$ for all $i=0,\dots ,m$. Moreover, and without loss of generality, we assume that $\varGamma$ and the Lipschitz partition $\left( \varOmega _j \right) _{ j=0\ldots n}$ are such that $\varOmega _0$ is the only unbounded domain.

Next, let us recall the notation ${\varGamma }_i= \varGamma \cap \partial \varOmega _i$ for $i=0,\dots ,m$. With this, we state one more assumption that we will need in order to show the required condition number bound:

#### Assumption 2

Let $\varGamma$ be a multi-screen as in Assumption 1. We assume that for each $j=0,..,m$, the family of meshes $\{{\varGamma }_{jh}\}_{h\in \mathcal {H}}, h > 0$ of ${\varGamma }_j$:

- agree at the junction(s);
- are uniformly shape-regular, and locally quasi-uniform;
- satisfy the following local mesh condition: For each triangle $\tau _l\in {\varGamma }_{jh}$, we define the index set $J(l):= \lbrace k \in \lbrace 1, \cdots ,M\rbrace \,: \, \tau _l\cap supp(\varphi _k)\ne 0 \rbrace$, where $M:=dim(\mathcal {S}_{0}^{1,0}({\varGamma }_{jh}))$. Moreover, for each basis function $\varphi _k \in \mathcal {S}_{0}^{1,0}({\varGamma }_{jh})$, local quasi-uniformity gives us an associated mesh size $\hat{h}_k$ that satisfies
  $$\begin{aligned} \frac{1}{c_Q} \le \frac{\hat{h}_k}{h_l} \le c_Q, \quad \forall \, l \text { such that } \tau _l\cap supp(\varphi _k)\ne 0, \, k=1,\ldots ,M, \end{aligned}$$
  where $h_l$ is the mesh size of $\tau _l$. Then, we require that
  33 $$\begin{aligned} \dfrac{57}{7} - \sqrt{ \sum _{k_1\in J(l)} \hat{h}_{k_1} \sum _{k_2\in J(l)} \hat{h}_{k_2}^{-1}} \ge c_0^j >0 \quad \forall \tau _l \in {\varGamma }_{jh}, \end{aligned}$$
  with a global constant $c_0^j>0$.

#### Remark 6

The local mesh condition (33) was first introduced in [30, Chapter 2.1]. It is considered mild because it is fulfilled by a broad set of meshes used in applications, including geometrically graded meshes, algebraically 2-graded meshes and families of meshes generated by adaptive red-green algorithms [18].

#### Remark 7

It is worth noticing that the local mesh condition (33) also guarantees that the $L^2(\varGamma )$-duality product between $\mathbb {X}_h(\varGamma )$ and $\mathbb {Y}_h(\varGamma )$ as chosen in (29) and (30) is stable [30]. Hence, by choosing $\textsf{m}$ to be $\ll \cdot , \cdot \gg$, our implementation leads to a Galerkin matrix $\textbf{M}_{{h}}$ that is bounded and invertible.

Under these considerations, we will show the following bound for the resulting condition number:

#### Theorem 1

Let $\textbf{W}_{h}$ be the Galerkin matrix defined in (31), and $\textbf{M}_{{h}}$ the Galerkin matrix of the duality pairing for $\mathbb {X}_h(\varGamma )\times \mathbb {Y}_h(\varGamma )$ as chosen above.

Assume that there exists an operator $\textsf{R}_h^+ \,: \, \mathbb {H}^{+\frac{1}{2}}(\varGamma )\rightarrow \mathbb {X}_h(\varGamma )$ such that

- $\textsf{R}_h^+$ is a h-uniformly bounded projection
- $$\textsf{R}_h^+ ( H^{+\frac{1}{2}}([\varGamma ])) \subseteq X_h(\varGamma ). $$

Then, under the mesh conditions from Assumption 2, we have

34 $$\begin{aligned} {\widetilde{\kappa }}_{sp}(\textbf{M}_{{h}}^{-1} \textbf{B}_{h}^{\textsf{V}}\textbf{M}_{{h}}^{-T}\textbf{W}_{h}) \le (1 +\vert \log h\vert )^2\dfrac{{\Vert \textsf{R}_h^+ \Vert } \alpha _{\textsf{M}}^2\Vert \textsf{W}_{0} \Vert \Vert \textsf{V}_{0} \Vert }{\Vert \textsf{M}\Vert ^2 \alpha _{\textsf{B}} \alpha _{\textsf{a}}}, \end{aligned}$$

where $\widetilde{\kappa }_{sp}$ denotes the spectral condition number away from the kernel, $\Vert \cdot \Vert$ the operator norms and $\alpha _{ {(\cdot )}}$ the corresponding inf-sup constants.

The proof will be given at the end of this Section, after we have presented some required preliminary results.

#### Remark 8

We refer the reader to [1, Theorem 2.1] for a construction of the operator $\textsf{R}_h^{+}$ for the case stated in Theorem 1. It is worth mentioning that here we still state it as a requirement to make it explicit that this is a key piece in our theory. Although we believe one can follow the cue from [1] to also build the projection operator required for $\textsf{V}_0$, the reader should be cautioned that story becomes considerably more complicated when considering Maxwell equations.

#### Remark 9

The existence of the operator $\textsf{R}_h^+$ required by Theorem 1 is proven in [1] for meshes that agree on the *front* and *back* of the structure. In practice this does not pose a major limitation because in a typical usage scenario the simple screens ${\varGamma _j}$ are built by fusing together two or more unique meshes for the interfaces $\partial \varOmega _i \cup \partial \varOmega _j$ (see Sect. 4.2). The interface meshes are used both as front and back and thus necessarily agree.

#### Auxiliary Lemmas

In this section we prove some auxiliary results that hold for the multi-screens under consideration. We remark that for this we follow the cue from [9, Sect. 5.2] and use the properties of the associated volume-based spaces.

##### Lemma 3

Let $\varGamma$ be a multi-screen as in Assumption 1. Then, there exist continuous embeddings

$$\begin{aligned} \mathbb {H}^{+\frac{1}{2}}(\varGamma )\hookrightarrow   H^{\frac{1}{2}}({\varGamma }_0)\times \dots \times H^{\frac{1}{2}}({\varGamma }_m), \\ \mathbb {H}^{- \frac{1}{2}}(\varGamma )\hookrightarrow   H^{-\frac{1}{2}}({\varGamma }_0)\times \dots \times H^{-\frac{1}{2}}({\varGamma }_m). \end{aligned}$$

##### Proof

We recall that $\mathbb {H}^{+\frac{1}{2}}(\varGamma )= H^1({\mathbb {R}^3}\backslash \overline{\varGamma })/ H^1_{0, \varGamma }({\mathbb {R}^3})$ and note that $H^1({\mathbb {R}^3}\backslash \overline{\varGamma }) \subset H^1({\mathbb {R}^3}\backslash \overline{\cup _{j=0}^m \partial \varOmega _j})$. This induces the injection

$$\begin{aligned} \mathbb {H}^{+\frac{1}{2}}(\varGamma )= H^1({\mathbb {R}^3}\backslash \overline{\varGamma })/ H^1_{0,\varGamma }( {\mathbb {R}^3}) \hookrightarrow   H^1({\mathbb {R}^3}\backslash \overline{\cup _{j=0}^m \partial \varOmega _j})/H^1_{0,\varGamma }({\mathbb {R}^3}). \end{aligned}$$

Additionally, we have the natural identification

$$\begin{aligned} H^1({\mathbb {R}^3}\backslash \overline{\cup _{j=0}^m \partial \varOmega _j}) \cong H^1(\varOmega _0) \times \dots H^1(\varOmega _m), \end{aligned}$$

that associates $u \in H^1({\mathbb {R}^3}\backslash \overline{\cup _{j=0}^m \partial \varOmega _j})$ with $(u_{\vert \varOmega _0}, \dots , u_{\vert \varOmega _m})$. From this natural identification, we get the isomorphism

$$\begin{aligned} H^1({\mathbb {R}^3}\backslash \overline{\cup _{j=0}^m \partial \varOmega _j})/H^1_{0,\varGamma }({\mathbb {R}^3})&\cong [H^1(\varOmega _0)/H^1_{0,\varGamma }(\varOmega _0)]\times \dots \times [H^1(\varOmega _m)/H^1_{0,\varGamma }(\varOmega _m)]\\&\cong H^{\frac{1}{2}}({\varGamma }_0)\times \dots \times H^{\frac{1}{2}}({\varGamma }_m). \end{aligned}$$

Therefore, we have the injection

$$\begin{aligned} \mathbb {H}^{+\frac{1}{2}}(\varGamma )\hookrightarrow   H^{\frac{1}{2}}({\varGamma }_0)\times \dots \times H^{\frac{1}{2}}({\varGamma }_m). \end{aligned}$$

$$\mathbb {H}^{- \frac{1}{2}}(\varGamma )\hookrightarrow   H^{-\frac{1}{2}}({\varGamma }_0)\times \dots \times H^{-\frac{1}{2}}({\varGamma }_m)$$ follows analogously. $$\square $$

##### Lemma 4

Let $\varGamma$ be a multi-screen as in Assumption 1. Then, for $w \in \mathbb {H}^{+\frac{1}{2}}(\varGamma )$ and $\varphi \in \mathbb {H}^{- \frac{1}{2}}(\varGamma )$ such that $\varphi _{\vert {\varGamma }_j} \in \widetilde{H}^{-\frac{1}{2}}({\varGamma }_j) \, \forall \, j=0,\dots ,m$, we have that

35 $$\begin{aligned} \ll w , \varphi \gg = \sum _{l=0}^m \langle w_{\vert {\varGamma }_l}, \varphi _{\vert {\varGamma }_l} \rangle _{S_l}, \end{aligned}$$

##### Proof

Let us consider $w \in \mathbb {H}^{+\frac{1}{2}}(\varGamma )$ and $\varphi \in \mathbb {H}^{- \frac{1}{2}}(\varGamma )$. By definition

$$\begin{aligned} \ll w, \varphi \gg = \int \nolimits _{[\varGamma ]} w \varphi \ d\sigma = \int _{{\mathbb {R}^3} \backslash \overline{\varGamma }} \textbf{p} \cdot \nabla U + U \textrm{div}(\textbf{p}) \ d\textbf{x}, \end{aligned}$$

for $U\in H^1({\mathbb {R}^3}\setminus \overline{\varGamma })$ and $\textbf{p} \in \textbf{H}(\textrm{div}, {\mathbb {R}^3}{\setminus }\overline{\varGamma })$ such that $\pi _D(U)=w$ and $\pi _N(\textbf{p}) = \varphi$.

For $j=0,\dots ,m$, we set $U_j = U_{\vert \varOmega _j}$ and $\textbf{p}_j = \textbf{p}_{\vert \varOmega _j}$, and let $\textbf{n}_j$ denote the outwards unit normal vector to $\varOmega _j$. Then, by linearity of the integrals and Green’s formula, we get

36 $$\begin{aligned} \int _{{\mathbb {R}^3} \backslash \overline{\varGamma }} \textbf{p} \cdot \nabla U + U \textrm{div}(\textbf{p}) \ d\textbf{x}&= \sum _{j=0}^m \, \int _{\varOmega _j} \textbf{p}_j \cdot \nabla U_j + U_j \textrm{div}(\textbf{p}_j) \ d\textbf{x}, \nonumber \\&= \sum _{j=0}^m \, \int _{\partial \varOmega _j} (U_{j})_{\vert \partial \varOmega _j} \textbf{n}_j \cdot (\textbf{p}_{j})_{\vert \partial \varOmega _j} d\sigma . \end{aligned}$$

Now, let us point out that for any $j=0,\dots , m$ we know that functions in $H^1 ({\mathbb {R}^3}{\setminus } \overline{\varGamma })$ and $\textbf{p} \in \textbf{H} (\textrm{div}, {\mathbb {R}^3}{\setminus }\overline{\varGamma })$ do not jump across $\partial \varOmega _j \setminus \varGamma$. This allow us to simplify (36) further as

37 $$\begin{aligned} \sum _{j=0}^m \, \int _{\partial \varOmega _j} (U_{j})_{\vert \partial \varOmega _j} \textbf{n}_j\cdot (\textbf{p}_{j})_{\vert \partial \varOmega _j} d\sigma = \int _{\varGamma } \sum _{j=0}^m v_j \mu _j d\sigma , \end{aligned}$$

where $$v_j = (U_j)_{\vert \varGamma }$$ and $$\mu _j = \textbf{n}_j \cdot (\textbf{p}_{j})_{\vert \varGamma }$$.

Next, we note that

$$\begin{aligned} \mu _j = \textbf{n}_j \cdot (\textbf{p}_{j})_{\vert \partial \varOmega _j} = \pi _N( \textbf{p}_{\vert \varOmega _j}) = (\pi _N( \textbf{p}) )_{\vert {\varGamma }_j} = \varphi _{ \vert {\varGamma }_j} \in \widetilde{H}^{-\frac{1}{2}}({\varGamma }_j),\\ v_j = (U_{j})_{\vert \partial \varOmega _j} = \pi _D( U_{\vert \varOmega _j}) = (\pi _D(U) )_{\vert {\varGamma }_j} = w_{\vert {\varGamma }_j} \in H^{\frac{1}{2}}({\varGamma }_j). \end{aligned}$$

This implies that on the right hand side of (37), we are allowed to split the integral over $\varGamma$ into the sum of the integrals over ${\varGamma }_j$ for $j=0,\dots ,m$. Furthermore, we can write it in terms of the $H^{\frac{1}{2}} ({\varGamma }_j) \times \widetilde{H}^{-\frac{1}{2}}({\varGamma }_j)$-duality pairings, i.e.

$$\begin{aligned} \int _{\varGamma } \sum _{j=0}^m v_j \mu _j d\sigma = \sum _{j=0}^m \int _{{\varGamma }_j} v_j \mu _j d\sigma = \sum _{j=0}^m \langle v_j, \mu _j \rangle _{{\varGamma }_j}. \end{aligned}$$

Finally, using again that $\mu _j = \varphi _{\vert {\varGamma }_j}$ and $v_j = w_{\vert {\varGamma }_j}$, we conclude that

$$\begin{aligned} \ll w, \varphi \gg _{\varGamma } = \sum _{j=0}^m \langle w_{\vert {\varGamma }_j}, \varphi _{ \vert {\varGamma }_j} \rangle _{{\varGamma }_j}. \end{aligned}$$

$$\square $$

The next ingredient we need is an inverse inequality on $\mathbb {H}^{+\frac{1}{2}}(\varGamma )$. Before we can derive it, we recall an inverse inequality on standard trace spaces. Let us consider the simple screens ${\varGamma }_j$ for $j=0,\dots ,m$ and let $\mathcal {S}^{0,-1} ({{\varGamma }}_{jh})$ be the space of piecewise constants on the mesh ${{\varGamma }}_{jh}$ of ${\varGamma }_j$.

##### Lemma 5

([21, Lemma 2.8], [34, Sect. 5], [33, Chap. 4]) For $j=0,\dots ,m$, the following inverse inequality holds:

38 $$\begin{aligned} \Vert \varphi _h\Vert _{\widetilde{H}^{-\frac{1}{2}}({\varGamma }_j)}&\le c_2(1+\vert \log h\vert )\Vert \varphi _h\Vert _{H^{-\frac{1}{2}}({\varGamma }_j)}, \end{aligned}$$

for all $\varphi _h\in \mathcal {S}^{0,-1}({\varGamma }_{jh})\subset \widetilde{H}^{-\frac{1}{2}}({\varGamma }_j)$, with mesh size $h\le 1$ and $c_2>0$ independent of *h*.

##### Remark 10

Lemma 5 also holds for all $\varphi _h\in \mathcal {S}^{0,-1}(\check{\varGamma }_{jh})\subset \widetilde{H}^{-\frac{1}{2}}({\varGamma }_j)$. In order to see this, one should note that: (i) all steps in the proof are valid for barycentric refinements; (ii) elements of $\mathcal {S}^{0,-1}(\check{\varGamma }_{jh})$ are linear combinations of piecewise constants on the barycentric refinement; (iii) our mesh assumptions guarantee that the number of neighbours is uniformly bounded with respect to the mesh size *h*. Hence, the coefficients of the linear combination can be absorbed by the constant $c_2$, which remains independent of *h*.

Let $\mathcal {S}^{1,0}({\varGamma }_{jh})$ be the space of piecewise linear functions on the primal mesh of ${\varGamma }_j$, as defined in [4]. We introduce the generalised $L^2$-projection $\tilde{Q}_h^j: L^2({\varGamma }_j) \rightarrow \mathcal {S}^{1,0}({\varGamma }_{jh})$ as

39 $$\begin{aligned} \langle \tilde{Q}_h^j u, \phi _{h}\rangle _{{\varGamma }_j} = \langle u, \phi _{h} \rangle _{{\varGamma }_j}, \qquad \forall \phi _{h} \in \mathcal {S}^{0,-1}({\check{\varGamma }}_{jh}). \end{aligned}$$

Then, from [23, Theorem 4.3], we know that under Assumption 2, we have

40 $$\begin{aligned} \Vert \tilde{Q}_h^j u\Vert _{H^{\frac{1}{2}}({\varGamma }_j)} \le c_{Qj} \Vert u \Vert _{ H^{\frac{1}{2}}({\varGamma }_j)}, \qquad \forall u \in H^{\frac{1}{2}}({\varGamma }_j). \end{aligned}$$

Moreover, [30, Thm. 2.1 and 2.2] shows that Assumption 2 implies the discrete inf-sup condition of the duality pairing of multi-trace spaces.

Next, note that $\mathbb {X}_h(\varGamma )$ and $\mathbb {Y}_h(\varGamma )$ defined in Sect. 4.2 generalize to^4^

41 $$\begin{aligned} \mathbb {X}_h(\varGamma )= \bigotimes _{j=0}^{m} \mathcal {S}_{0}^{1,0}(\varGamma _{jh}), \quad \text { and } \quad \mathbb {Y}_h(\varGamma )= \bigotimes _{j=0}^{m} \mathcal {S}^{0,-1}(\check{\varGamma }_{jh}). \end{aligned}$$

##### Lemma 6

(Inverse inequality in $\mathbb {H}^{- \frac{1}{2}}(\varGamma )$) Let $\varGamma$ be a multi-screen as in Assumption 1 and consider finite dimensional spaces $\mathbb {X}_h(\varGamma )\subset \mathbb {H}^{+\frac{1}{2}}(\varGamma )$ and $\mathbb {Y}_h(\varGamma )\subset \mathbb {H}^{- \frac{1}{2}}(\varGamma )$ defined in (41) and such that Assumption 2 is satisfied. Then, we have that for all $\varphi _h \in {\mathbb {Y}_h(\varGamma )}$

42 $$\begin{aligned} \Vert \varphi _h \Vert _{\mathbb {H}^{- \frac{1}{2}}(\varGamma )} \le {C_P} (1 + \vert \log h \vert ) \left( \sum _{j=0}^m \Vert \varphi _h \Vert _{H^{-\frac{1}{2}}({\varGamma }_j)}^2 \right) ^{1/2}, \end{aligned}$$

with mesh size $h\le 1$, and ${C_P}>0$ independent of *h*.

##### Proof

By definition of dual norm and Lemma 4, we get

43 $$\begin{aligned} \Vert \varphi _h \Vert _{\mathbb {H}^{- \frac{1}{2}}(\varGamma )} = \underset{v\in \mathbb {H}^{+\frac{1}{2}}(\varGamma )\setminus \lbrace 0\rbrace }{\sup } \dfrac{\vert \ll v,\varphi _h\gg \vert }{\Vert v \Vert _{\mathbb {H}^{+\frac{1}{2}}(\varGamma )}} = \underset{v\in \mathbb {H}^{+\frac{1}{2}}(\varGamma )\setminus \lbrace 0\rbrace }{\sup }\dfrac{\vert \sum _{j=0}^m \langle v_j, \varphi _{hj} \rangle _{{\varGamma }_j} \vert }{\Vert v \Vert _{\mathbb {H}^{+\frac{1}{2}}(\varGamma )}}, \end{aligned}$$

where we have set $v_j = v_{\vert {\varGamma }_j}$ and $\varphi _{hj} = (\varphi _h)_{\vert {\varGamma }_j}$. Then, let us consider the index set $\mathcal {J}$ of all the indices $0\le k \le m$ such that $v_k \ne 0$. Thus, we have that

44 $$\begin{aligned} \dfrac{\vert \sum _{j=0}^m \langle v_j, \varphi _{hj} \rangle _{{\varGamma }_j} \vert }{\Vert v \Vert _{\mathbb {H}^{+\frac{1}{2}}(\varGamma )}} = \dfrac{\vert \sum _{j\in \mathcal {J}} \langle v_j, \varphi _{hj} \rangle _{{\varGamma }_j} \vert }{\Vert v \Vert _{\mathbb {H}^{+\frac{1}{2}}(\varGamma )}}. \end{aligned}$$

Next, we derive two inequalities that will help us proceed. First, using the embedding from Lemma 3 and then Young’s inequality *m*-times, we obtain

45 $$\begin{aligned} \Vert v \Vert ^2_{\mathbb {H}^{+\frac{1}{2}}(\varGamma )} \ge \sum _{j\in \mathcal {J}} \Vert v_j \Vert ^2_{ H^{\frac{1}{2}}({\varGamma }_j)} \ge \dfrac{1}{\vert \mathcal {J} \vert } \left( \sum _{j\in \mathcal {J}} \Vert v_j \Vert _{H^{\frac{1}{2}}({\varGamma }_j)} \right) ^2 \end{aligned}$$

where $\vert \mathcal {J} \vert$ is the size of the index set $\mathcal {J}$. Second, we remark that for a sum $\sum _{i=1}^{m^*} a_i$ with all coefficients $a_i >0$ and $m^*\in \mathbb {N}$, we have that $\dfrac{1}{\sum _{i=1}^{m^*} a_i} \le \dfrac{1}{a_k}$ for all $k=1,\dots m^*$. Hence,

46 $$\begin{aligned} \dfrac{m^*}{\sum _{i=1}^{m^*} a_i} \le \sum _{k=1}^{m^*} \dfrac{1}{a_k} \end{aligned}$$

Plugging these in (43) gives

47 $$\begin{aligned} \Vert \varphi _h \Vert _{\mathbb {H}^{- \frac{1}{2}}(\varGamma )}&\overset{(44)}{=} \underset{v\in \mathbb {H}^{+\frac{1}{2}}(\varGamma )\setminus \lbrace 0\rbrace }{\sup }\dfrac{\vert \sum _{j\in \mathcal {J}} \langle v_j, \varphi _{hj} \rangle _{{\varGamma }_j} \vert }{\Vert v \Vert _{\mathbb {H}^{+\frac{1}{2}}(\varGamma )}} \nonumber \\&\overset{(45)}{\le } \vert \mathcal {J}\vert ^{1/2} \underset{v\in \mathbb {H}^{+\frac{1}{2}}(\varGamma )\setminus \lbrace 0\rbrace }{\sup }\dfrac{\vert \sum _{j\in \mathcal {J}} \langle v_j,\varphi _{hj}\rangle _{{\varGamma }_j}\vert }{\sum _{k\in \mathcal {J}} \Vert v_k \Vert _{H^{\frac{1}{2}} (S_k)}} \nonumber \\ \,&\overset{(46)}{\le } \dfrac{1}{\vert \mathcal {J}\vert ^{1/2}} \underset{v\in \mathbb {H}^{+\frac{1}{2}}(\varGamma )\setminus \lbrace 0\rbrace }{\sup }\vert \sum _{j\in \mathcal {J}} \dfrac{\langle v_j, \varphi _{hj}\rangle _{{\varGamma }_j}}{\Vert v_j \Vert _{H^{\frac{1}{2}} ({\varGamma }_j)}} \vert \nonumber \\ \,&\le \dfrac{1}{\vert \mathcal {J}\vert ^{1/2}} \sum _{j\in \mathcal {J}} \, \underset{w_j\in H^{\frac{1}{2}}({\varGamma }_j)\setminus \lbrace 0\rbrace }{\sup } \dfrac{\vert \langle w_j, \varphi _{hj}\rangle _{{\varGamma }_j}\vert }{\Vert w_j \Vert _{H^{\frac{1}{2}}({\varGamma }_j)}} \nonumber \\&= \dfrac{1}{\vert \mathcal {J}\vert ^{1/2}} \sum _{j=0}^{m} \, \underset{w_j\in H^{\frac{1}{2}}({\varGamma }_j)\setminus \lbrace 0\rbrace }{\sup } \dfrac{\vert \langle w_j, \varphi _{hj}\rangle _{{\varGamma }_j}\vert }{\Vert w_j \Vert _{H^{\frac{1}{2}} ({\varGamma }_j)}}. \end{aligned}$$

Then, using $\tilde{Q}_h^j$ from (39) and its continuity (40), we get

48 $$\begin{aligned} \Vert \varphi _h \Vert _{\mathbb {H}^{- \frac{1}{2}}(\varGamma )}&\le \dfrac{1}{\vert \mathcal {J}\vert ^{1/2}} \sum _{j=0}^{m} \, c_{Qj} \underset{w_j\in H^{\frac{1}{2}}({\varGamma }_j)\setminus \lbrace 0\rbrace }{\sup } \dfrac{\vert \langle \tilde{Q}_h^j w_j, \varphi _{hj}\rangle _{{\varGamma }_j}\vert }{\Vert \tilde{Q}_h^j w_j \Vert _{H^{\frac{1}{2}} ({\varGamma }_j)}} \nonumber \\&\le \dfrac{1}{\vert \mathcal {J}\vert ^{1/2}} \sum _{j=0}^{m} \, c_{Qj} \underset{w_{hj}\in \mathcal {S}^{0,1}({\varGamma }_{jh}) \setminus \lbrace 0\rbrace }{\sup } \dfrac{\vert \langle w_{hj}, \varphi _{hj} \rangle _{{\varGamma }_j}\vert }{\Vert w_{hj} \Vert _{H^{\frac{1}{2}} ({\varGamma }_j)}}. \end{aligned}$$

Applying Cauchy-Schwarz and (38), we obtain

49 $$\begin{aligned} \Vert \varphi _h \Vert _{\mathbb {H}^{- \frac{1}{2}}(\varGamma )}&\le \dfrac{1}{\vert \mathcal {J}\vert ^{1/2}} \sum _{j=0}^{m} \, c_{Qj} \Vert \varphi _{hj} \Vert _{\widetilde{H}^{-\frac{1}{2}} ({\varGamma }_j)} \nonumber \\&\le {c_3}(1 + \vert \log h\vert ) \sum _{j=0}^{m} \, \Vert \varphi _{hj} \Vert _{ H^{-\frac{1}{2}} ({\varGamma }_j)}, \end{aligned}$$

with ${c_3}:= \dfrac{c_2 \max (c_{Qj})}{\vert \mathcal {J}\vert ^{1/2}}$. For convenience, we work the estimate further

50 $$\begin{aligned} \Vert \varphi _h \Vert _{\mathbb {H}^{- \frac{1}{2}}(\varGamma )}^2&\le {c_3}^2 (1 + \vert \log h\vert )^2 \left( \sum _{j=0}^{m}\, \Vert \varphi _{hj}\Vert _{H^{-\frac{1}{2}}({\varGamma }_j)}\right) ^2 \nonumber \\  &\, \overset{(45)}{\le }\vert \mathcal {J}\vert {c_3}^2 (1 + \vert \log h\vert )^2 \sum _{j=0}^{m} \, \Vert \varphi _{hj} \Vert _{H^{-\frac{1}{2}} ({\varGamma }_j)}^2, \end{aligned}$$

which gives (42) with ${C_P} = c_2\max (c_{Qj})$.

$$\square $$

Finally, recall that

$$\begin{aligned} \Vert u \Vert _{\widetilde{H}^{+\frac{1}{2}}([\varGamma ])} = \inf _{x \in H^{+\frac{1}{2}}([\varGamma ])} \Vert u + x \Vert _{\mathbb {H}^{+\frac{1}{2}}(\varGamma )}, \end{aligned}$$

and that $\mathcal {S}^{1,0}(\mathcal {T}_h)$ is the space spanned by piecewise linear “continuous” functions on (the virtual mesh) $\mathcal {T}_h$. We define the norm

51 $$\begin{aligned} \Vert u \Vert _{\widetilde{H}^{+\frac{1}{2}}([\varGamma ]),D}:= \inf _{x_h \in H^{+\frac{1}{2}}([\varGamma ])\cap \mathcal {S}^{1,0}(\mathcal {T}_h)} \Vert u + x_h \Vert _{\mathbb {H}^{+\frac{1}{2}}(\varGamma )}, \end{aligned}$$

and study its relation with the continuous jump norm.

##### Lemma 7

Assume that there exists an operator $\textsf{R}_h^+ \,: \, \mathbb {H}^{+\frac{1}{2}}(\varGamma )\rightarrow \mathcal {S}^{ 1,0}(\mathcal {T}_h)$ such that

(i) $\textsf{R}_h^+$ is a h-uniformly bounded projection,
(ii) $$\textsf{R}_h^+(H^{+\frac{1}{2}}([\varGamma ])) \subseteq H^{+\frac{1}{2}}([\varGamma ])\cap \mathcal {S}^{1,0}(\mathcal {T}_h).$$

Then there exist constants $c_e, C_e>0$ independent of *h* and such that

$$\begin{aligned} c_e \Vert u_h \Vert _{\widetilde{H}^{+\frac{1}{2}}([\varGamma ])} \le \Vert u_h \Vert _{\widetilde{H}^{+\frac{1}{2}}([\varGamma ]),D} \le C_e \Vert u_h \Vert _{\widetilde{H}^{+\frac{1}{2}}([\varGamma ])},\quad \forall u_h \in \widetilde{H}^{+\frac{1}{2}}([\varGamma ])\cap \mathcal {S}^{1,0}(\mathcal {T}_h). \end{aligned}$$

##### Proof

By definition, for $u_h \in \widetilde{H}^{+\frac{1}{2}}([\varGamma ])\cap \mathcal {S}^{1,0}(\mathcal {T}_h)$, we have that

52 $$\begin{aligned} \Vert u_h \Vert _{\widetilde{H}^{+\frac{1}{2}}([\varGamma ])} \le \Vert u_h \Vert _{\widetilde{H}^{+\frac{1}{2}}([\varGamma ]),D} \end{aligned}$$

Choose $u_h = \textsf{R}_h^+ u$ where $u\in \widetilde{H}^{+\frac{1}{2}}([\varGamma ])\subset \mathbb {H}^{+\frac{1}{2}}(\varGamma )$. Then we get

$$\begin{aligned} \Vert u_h \Vert _{\widetilde{H}^{+\frac{1}{2}}([\varGamma ]),D} = \inf _{x_h \in H^{+\frac{1}{2}}([\varGamma ])\cap \mathcal {S}^{1,0}(\mathcal {T}_h)} \Vert u_h + x_h \Vert _{\mathbb {H}^{+\frac{1}{2}}(\varGamma )}. \end{aligned}$$

Note that, by surjectivity of $\textsf{R}_h^+$ and property (ii), there exists an $x \in H^{+\frac{1}{2}}([\varGamma ])$ such that $x_h = \textsf{R}_h^+ x$. This allows us to write

$$\begin{aligned} \Vert u_h \Vert _{\widetilde{H}^{+\frac{1}{2}}([\varGamma ]),D} = \inf _{{x} \in H^{+\frac{1}{2}}([\varGamma ])} \Vert \textsf{R}_h^+(u+x) \Vert _{\mathbb {H}^{+\frac{1}{2}}(\varGamma )}. \end{aligned}$$

Since $\textsf{R}_h^+$ is continuous, we further obtain

53 $$\begin{aligned} \Vert u_h \Vert _{\widetilde{H}^{+\frac{1}{2}}([\varGamma ]),D} \le \Vert \textsf{R}_h^+ \Vert \inf _{{x} \in H^{+\frac{1}{2}}([\varGamma ])} \Vert u+x \Vert _{\mathbb {H}^{+\frac{1}{2}}(\varGamma )} = \Vert \textsf{R}_h^+ \Vert \Vert u_h \Vert _{\widetilde{H}^{+\frac{1}{2}}([\varGamma ])} \end{aligned}$$

for all $${u}_h \in \widetilde{H}^{+\frac{1}{2}}([\varGamma ])\cap \mathcal {S}^{1,0}(\mathcal {T}_h)$$.

From this, we conclude that the two norms are equivalent. Moreover, the fact that $\textsf{R}_h^+$ is *h*-uniformly bounded guarantees that the related constants are *h*-independent.

$$\square $$

#### Proof of Theorem 1

We are now in the position to show the ellipticity of our preconditioner and then prove Theorem 1.

##### Proposition 4

Let ${\textsf{B}_{h}}^{\textsf{V}} :\, \mathbb {Y}_h(\varGamma )\rightarrow (\mathbb {Y}_h(\varGamma ))^\prime$ be the linear operator corresponding to $\textbf{B}_{h}^{\textsf{V}}$ defined in (58). For the discrete spaces defined in this section, we have that for all $h {\in \mathbb {R}}$ it holds that

54 $$\begin{aligned} {\ll \textsf{B}_h^{\textsf{V}} u_h, u_h \gg \, \ge \alpha _{\textsf{B}_{\textsf{V}}} (1 + \vert \log h \vert )^{-2} \Vert u_h \Vert _{\mathbb {H}^{- \frac{1}{2}}(\varGamma )}^2} \end{aligned}$$

for all $u_h\in {\mathbb {H}^{- \frac{1}{2}}(\varGamma )}$, and with $\alpha _{\textsf{B}_{\textsf{V}}}>0$ independent of *h*.

##### Proof

By definition of $\textbf{B}_{h}^{\textsf{V}}$ we have that

55 $$\begin{aligned} \ll \textsf{B}_h^{\textsf{V}} u_h, u_h \gg \, = \sum _{l} \langle \textsf{V}_0 u_h, u_h \rangle _{ \mathcal {I}_{l}} \end{aligned}$$

Let us write $u_{hl}:= u_{h|\mathcal {I}_{l}}$. Recall that $\textsf{V}_{0}$ is elliptic on each $\mathcal {I}_l$ and hence

$$\begin{aligned} \langle \textsf{V}_{0} u_{hl}, u_{hl} \rangle _{\mathcal {I}_{l}} \ge \tilde{c}_l\Vert u_{hl} \Vert _{\widetilde{H}^{-\frac{1}{2}}(\mathcal {I}_{l})}^2 \ge \tilde{c}_l \Vert u_{hl} \Vert _{{H}^{-\frac{1}{2}}(\mathcal {I}_{l})}^2 \end{aligned}$$

is satisfied for all *h*, and with $\tilde{c}_l>0$ independent of *h*. Therefore, setting $c_*= \min \tilde{c}_l$, we get

56 $$\begin{aligned} \ll \textsf{B}_h^{\textsf{V}} u_h, u_h \gg \, \ge \sum _{l} \tilde{c}_l \Vert u_{hl} \Vert _{ {H}^{-\frac{1}{2}}(\mathcal {I}_{l})}^2 \ge c_* \sum _{l} \Vert u_{hl} \Vert _{{H}^{-\frac{1}{2}}( \mathcal {I}_{l})}^2. \end{aligned}$$

Finally, using the inverse inequality from Lemma 6, we obtain the desired result with $\alpha _{\textsf{B}_{\textsf{V}}}:= \dfrac{c_*}{\tilde{C}_{N}^2}$. $\square$

##### Proof of Theorem 1

Given the ellipticity constants from Propositions 2 and 4, our bilinear forms satisfy their inf-sup constants. From Lemma 7, norm equivalence holds with $\Vert u_h \Vert _{\mathbb {X}/X_h} \le \Vert \textsf{R}_h^+ \Vert \Vert u_h \Vert _{\mathbb {X}/X}$ for all $u_h$ in $\mathbb {X}_h$. This together with Lemma 2 gives that the result follows from the bound derived in Sect. 3. $\square$

## Calderón preconditioning on reduced quotient-space BEM

The above analysis has been carried out for the case where the BE space is chosen to approximate the entire multi-trace space. Since the solution is determined in the jump space, it can be worth while to investigate whether (combinations of) basis functions can be deleted and whether the resulting method remains amenable to operator preconditioning schemes.

In this section we will introduce several ways in which the number of basis functions can be reduced, what mileage can be expected from the resulting methods, and we discuss what the ramifications are for implementations in code of these methods.

The most straightforward approach to building a well-defined BEM formulation on multi-screens is to introduce a BE space for the multi-trace space that is contained in the product space. Two key ingredients for the success of this approach are that

- the discrete left/right nullspace $X_h(\varGamma )$ equals $H^{+\frac{1}{2}}([\varGamma ])\cap \mathbb {X}_h(\varGamma )$; and that
- the quotient $\mathbb {X}_h(\varGamma )/ X_h(\varGamma )$ approximates $\widetilde{H}^{+\frac{1}{2}}([\varGamma ])$.

However, because we are interested in finding an approximate solution in the quotient space $\widetilde{H}^{+\frac{1}{2}}([\varGamma ])$, we are free to consider BE spaces $\mathbb {X}_h(\varGamma )^\circ$ that do not approximate all of $\mathbb {H}^{+\frac{1}{2}}(\varGamma )$ as long as the corresponding discrete nullspace $X_h(\varGamma )^\circ =H^{+\frac{1}{2}}([\varGamma ])\cap \mathbb {X}_h(\varGamma )^\circ$ is still a subset of $H^{+\frac{1}{2}}([\varGamma ])\cap \mathbb {X}_h(\varGamma )$ and the quotient spaces $\mathbb {X}_h(\varGamma )^\circ / X_h(\varGamma )^\circ$ and $\mathbb {X}_h(\varGamma )/ X_h(\varGamma )$ are equal. Similar choices can be made to select a reduced dual BE space $\mathbb {Y}_h(\varGamma )^\circ \subseteq \mathbb {Y}_h(\varGamma )$. The quality of the resulting operator preconditioning depends on the stability of the restriction of the duality form to this subspace.

How does this work in practice? In the case of nodal elements in $\mathcal {S}_{ 0}^{1,0}(\mathcal {T}_h)$, any given basis function relates to a function in $X_h(\varGamma )$ by completing it with its counterpart(s) on the opposite side(s) of the multi-screen. By removing one of the basis functions from the standard nodal basis for $\mathbb {X}_h(\varGamma )^\circ$, the dimension of the discrete nullspace $X_h(\varGamma )^\circ$ decreases by one. The dimension of the complement of $X_h(\varGamma )^\circ$ remains unchanged and so necessarily $\mathbb {X}_h(\varGamma )^\circ / X_h(\varGamma )^\circ = \mathbb {X}_h(\varGamma )/ X_h(\varGamma )$. To put it in more physical terminology: the reduced discrete multi-trace space $\mathbb {X}_h(\varGamma )^\circ$ *radiates* the same fields as the original one.

There are a number of reduction schemes that are fairly straightforward to implement. We will discuss three strategies that can be applied to a multi-screen $\varGamma$ comprising a single junction where an odd number *m* simple screens meet.

(i) *Partial reduction*: In the partition $[\varGamma ] = \cup _{i=1}^{m} \mathcal {I}_i$, basis functions based on the terms $i=3,5,7,...$ can be discarded. This is extremely easy to implement and boils down to using $\mathbb {X}_h(\varGamma )^\circ = {\mathcal {S}_{0}^{1,0}}(\mathcal {I}_{1,h}) \times {\bigotimes }_{ i=1}^{\lfloor m/2 \rfloor } {\mathcal {S}_{0}^{1,0}}(\mathcal {I}_{2i,h})$ instead of $\mathbb {X}_h(\varGamma )= {\bigotimes }_{i=1}^m {\mathcal {S}_{0}^{1,0}}(\mathcal {I}_{i,h})$.
(ii) *Single strip*: The partial reduction described above in essence removes the *back* from part of $[\varGamma ]$. This still leaves significant redundancy in $\mathbb {X}_h(\varGamma )^\circ$. In our example leaving out ${\mathcal {S}_{0}^{1, 0}}(\mathcal {I}_{i,h})$ for $i=3,5,7,...$ still leaves all the basis functions on $\varGamma _2$ (excluding basis functions on the junction) that can be completed by basis functions on *the other side* to yield functions in $X_h(\varGamma )$. As a result, we can further discard basis functions in ${\mathcal {S}_{0}^{1,0}}(\mathcal {I}_{1,h})$ that lie in the interior of $\varGamma _2$. If the implementer has access to node-triangle adjacency information this approach requires minimal coding effort. The resulting BE space is $X_h(\varGamma )^\circ =\mathcal {S}_{0}^{1,0}(\mathcal {I}_{1,h})^\circ \times {\bigotimes }_{i=1}^{\lfloor m/2 \rfloor } {\mathcal {S}_{0}^{1,0}}(\mathcal {I}_{2i,h})$, where the $\circ$ superscript on the first factor denotes that this BE space is reduced by leaving out redundant basis functions linked to nodes that are in $\mathcal {I}_{1,h}\cap \varGamma _2$ but not on the junction.
(iii) *Fixed overlap*: Note that the efficiency of the resulting preconditioning method depends on the lower bound for the duality form $\ll .,.\gg$ on $\mathbb {X}_h(\varGamma )^\circ \times \mathbb {Y}_h(\varGamma )^\circ$, which may depend on the geometry and hence may indirectly depend on *h* when using a single strip reduction. In those situations where this is undesirable, one can opt to retain not only those basis functions in ${\mathcal {S}_{0}^{1,0}}(\mathcal {I}_{1,h})$ that are positioned on $\varGamma _1$ or on the junction, but also those on $\varGamma _2$ inside a strip within a fixed mesh independent distance from the junction. Likely, this will require manipulations to the code at the level of mesh generation. The resulting method will lead to an increasing redundancy in basis functions as *h* tends to zero, but the user is guaranteed that the preconditioner efficiency will not be limited by degradation of the stability of the duality pairing.

**Fig. 7:**
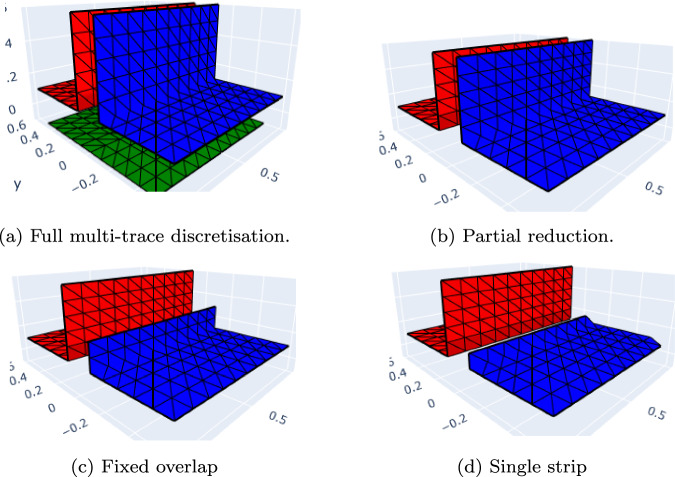
Meshes illustrating full multi-trace discretisation and three reduction strategies used here

We illustrate these three reduction strategies for $m=3$ on Fig. 7. We also point out that when *m* is even, all these reductions are also valid, but since one can always find a *partial reduction* that provides a minimal representation of the quotient space, i.e. $\mathbb {X}_h(\varGamma )^\circ = {\bigotimes }_{i=1}^{m/2} {\mathcal {S}_{0}^{1,0}}(\mathcal {I}_{2i, h})$, the other two proposed strategies are not computationally attractive.

Finally, it is worth mentioning that regardless the choice of reduction method and the corresponding primal BE space $\mathbb {X}_h(\varGamma )^\circ$, the construction of the dual BE space $\mathbb {Y}_h(\varGamma )^\circ$ remains the same. The construction goes along the lines of what is described in [4], starting from the reduced surfaces and corresponding meshes

57 $$\begin{aligned} \mathcal {I}_i \cap \bigcup _{u \in \mathbb {X}_h(\varGamma )^\circ } {\text {supp}} u \end{aligned}$$

This means in particular that for the Neumann problem, the dual space simply consists of piecewise constants on the dual cells as detailed in [3].

Moreover, since the discrete stability of the duality pairing also implies the continuity estimate (40) used in our proofs, we have that by ensuring this stability, all results in Sect. 4 can be extended to the proposed reduced Quotient-space BEM and hence we are still within the framework of Theorem 1. For this, it is crucial to identify the reduced primal space on $\mathcal {I}_{1,h}$ with the (full) space on a truncated simple screen $\mathcal {I}_{1,h}^\circ$, which are displayed in blue in Fig. 7c and d. So for example, one identifies ${\mathcal {S}_{0}^{1,0}}(\mathcal {I}_{1,h})^\circ$ with ${\mathcal {S}_{0}^{ 1,0}}(\mathcal {I}_{1,h}^\circ )$.

## Numerical results

### Preconditioning the hypersingular operator (Neumann problem)

Consider the geometry in Fig. 7. The structure is submersed in an externally generated potential $u^{inc}(x) = x_1 + x_3$.

Linear systems are solved by the preconditioned conjugate gradient (PCG) method with the relative tolerance set to $2.0e-5$. To build the preconditioners, application of the inverse Gram matrix is required. This action is computed by running a second, inner GMRES solver (the Gram matrices are between different BE spaces and thus not self-adjoint) within the outer, primal solver. Numerical experiments have shown that it is important to set the tolerance for this inner GMRES sufficiently low. In the experiments presented here the tolerance is set to $2.0e-12$. Fortunately the Gram matrices are well conditioned and application of their inverses through GMRES can be computed in a small and linear number of operations, even for these very small tolerances.

Another important aspect of implementing the preconditioning strategies presented above is the use of high quality quadrature rules, especially for interactions between geometric elements that are close together. Specifically, it is important that left and right nullspaces of the discrete bilinear forms are invariant upon introduction of the quadrature error. One can either choose to adopt highly accurate quadrature rules or to use rules that are symmetric with respect to back-front mirroring across the multi-screen. Here we have opted for the highly accurate and kernel independent Sauter-Schwab rules described in [29, Chapter 5].

**Fig. 8:**
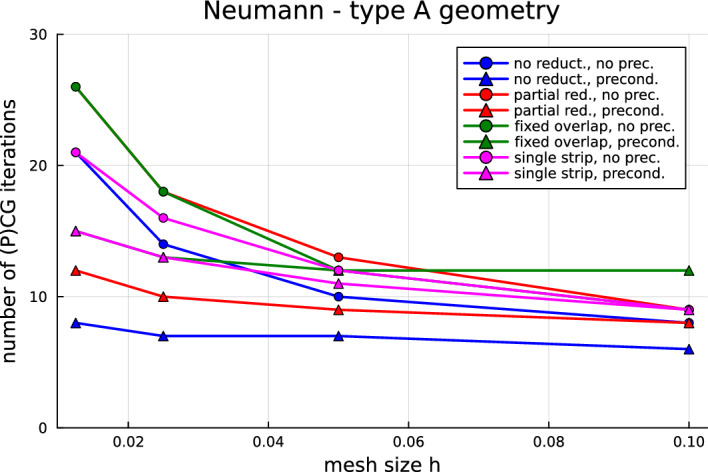
PCG iterations vs *h* for the Neumann problem at $\varGamma$ as in Fig. 7

The obtained results are displayed in Fig. 8. For all reductions of $\mathbb {X}_h(\varGamma )$ depicted in Fig. 7, there is a clear improvement. After preconditioning there remains a slow increase in the number of iterations, commensurate with the logarithmic grow in the (34).

### Preconditioning the weakly singular operator (Dirichlet problem)

We consider the same geometry, excitation and PCG tolerance used to study our preconditioner for the Neumann problem. An important difference is that basis functions for the Dirichlet problem are linked to triangles of the mesh, as opposed to vertices. The support of the primal basis functions spans only a single triangle. The reduction of the multi-trace space can be done up to the point where there is no overlap between the simple screens that support the reduced finite element spaces. For ease of comparison, we still refer to this reduction strategy as *single strip*.

To arrive at a non-singular preconditioner, the hypersingular operator is regularised as in [31], resulting in a block diagonal preconditioner based on

58 $$\begin{aligned} \textbf{B}_{h}^{\textsf{W}} := \left( \begin{array}{c c c} \check{\textbf{W}}^r_{h,1} &  \textbf{0} &  \textbf{0} \\ \textbf{0} & \check{\textbf{W}}^r_{h,2} &  \textbf{0} \\ \textbf{0} & \textbf{0} & \check{\textbf{W}}^r_{h,3}\\ \end{array}\right) , \end{aligned}$$

where $\check{\textbf{W}}^r_{h,l}[i,j]=\langle \textsf{W}_{0} \check{\psi }_j, \check{\psi }_i \rangle _{\mathcal {I}_{l}} + \langle \check{\psi }_j, 1_l \rangle _{\mathcal {I}_{l}} \langle 1_l, \check{\psi }_i \rangle _{\mathcal {I}_{l}}$ with $\check{\psi }_i,\check{ \psi }_j$ in the standard basis of $\mathcal {S}^{1,0}(\check{\mathcal {I}}_{l,h})$ for $l=1,2,3$ and $1_l$ the constant function on $\mathcal {I}_{l}$ taking on the value 1.

**Fig. 9:**
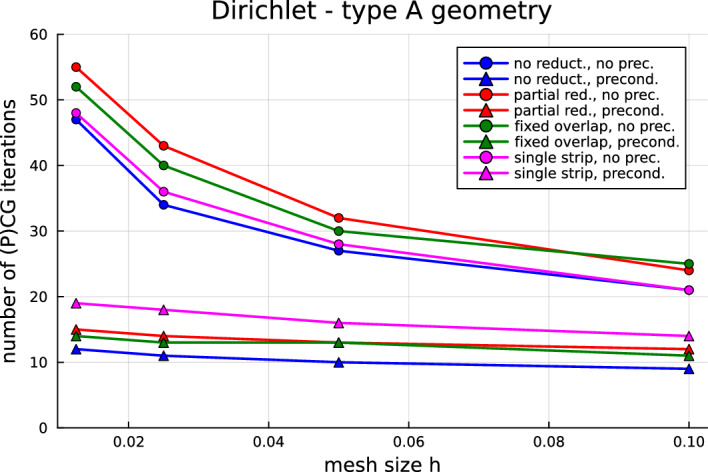
PCG iterations vs *h* for the Dirichlet problem at $\varGamma$ as in Fig. 7

Essentially all conclusions drawn for the Neumann problem carry over to the study of the numerical solution of the Dirichlet problem (Fig. 9): for all reduction strategies, application of the block diagonal Calderón preconditioner leads to a much smaller number of iterations.

### Application to multi-screens of Type B

**Fig. 10:**
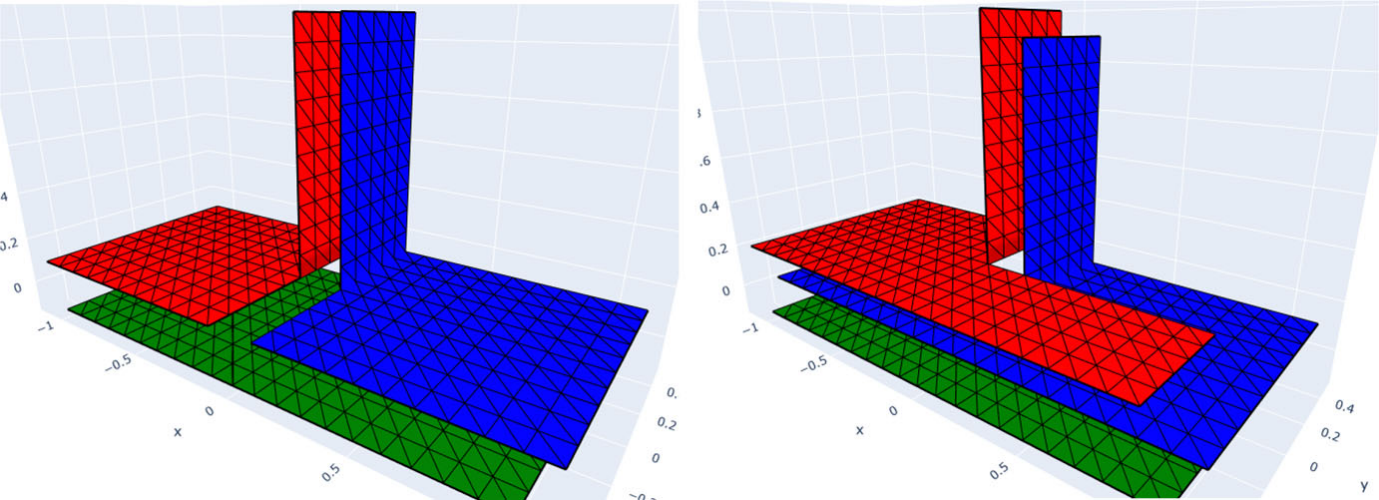
Two possible coverings for $[\varGamma ]$. The most economic covering on the left precludes the definition of BE spaces of direct product type (left). Allowing part of $[\varGamma ]$ to be multiply covered resolves this problem (right)

For multi-screens of Type B, slight modifications to the choice of BE spaces are required in order to arrive at a linear system requiring only few iterations for its solution. It may seem most natural to write $[\varGamma ]$ as the union of the simple screens depicted on the left in Fig. 10. Unfortunately, this partitioning does not allow the construction of a BE space $\mathbb {X}_h(\varGamma )$ that can be written as the product of BE spaces supported by the $\mathcal {I}_i$. The issue is that the solution for the Neumann problem in general will not be in ${\bigotimes }_i \widetilde{H}^{\frac{1}{2}}( \mathcal {I}_i)$ and, as a result, basis functions along the segment from (0, 0, 0) to $(0,-0.5,0)$ cannot be discarded.

Allowing overlapping coverings of $[\varGamma ]$ as depicted on the right of Fig. 10 solves this problem. For $l=1,..,m$, let $\bar{\mathcal {I}}_{l}$ denote either $\mathcal {I}_l$ with overlap when needed, or without overlap. We can use the BE space ${\bigotimes }_{i=1}^m \mathcal {S}^{1,0}(\bar{\mathcal {I}}_{i,h})$, which in a sense is larger than what we need but still leads to the correct quotient space.

**Fig. 11:**
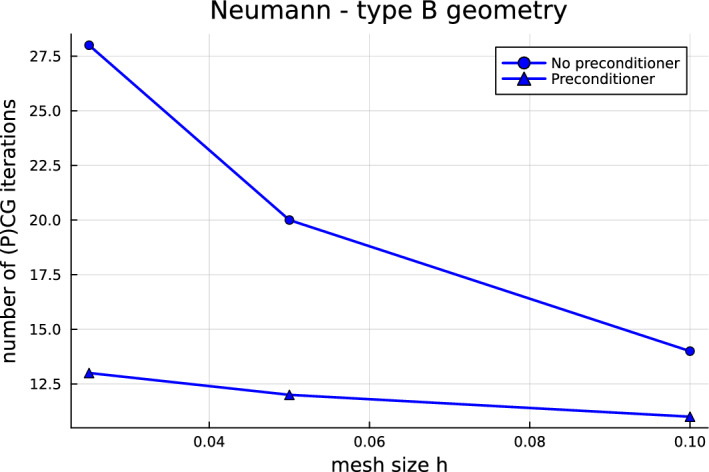
PCG iterations vs *h* for the Neumann problem for scattering by a geometry of type B

We use this approach to solve the Neumann problem for the geometry in Fig. 10 and for the excitation $u^{inc}(x) = x_1 + x_3$. Figure 11 shows that upon preconditioning the number of iterations is much lower than what is required to solve the original linear system when solving the Neumann problem.

**Fig. 12:**
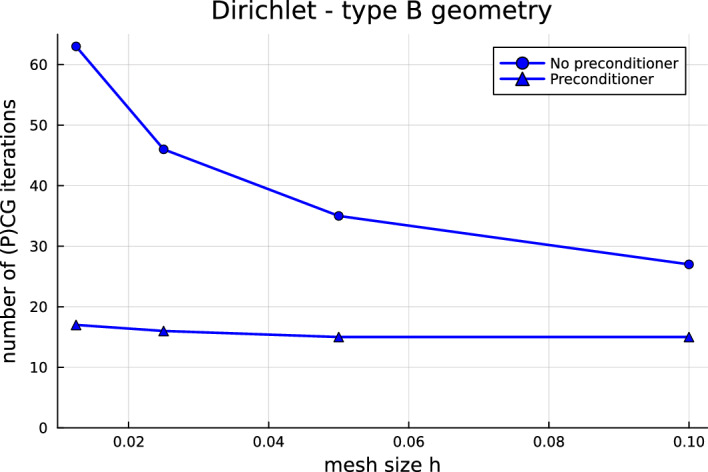
PCG iterations vs *h* for the Dirichlet problem for scattering by a geometry of type B

Figure 12 shows the results for the Dirichlet problem. They are in line with those from Fig. 9: the higher offset in the iteration count results in a cross-over point at smaller values of *h*, but asymptotically the preconditioner leads to a more efficient algorithm.

The numerical results presented in this section have been produced with the boundary element package BEAST.jl.^5^ The scripts to reproduce them can be found in a public Github repository.^6^

Note that this strategy, where we allow part of $[\varGamma ]$ to be multiply covered, can also be applied to enable the modelling of potential problems near Type C multi-screens such as the Möbius strip.

### Supplement: results for the Helmholtz equation

The above analysis does not hold verbatim for BIEs for the solution of Helmholtz BVPs: the possibility of resonances downgrades ellipticity estimates for the BIOs to mere Gårding inequalities and the number of required GMRES iterations is only loosely connected to the spectral condition number. Notwithstanding the method described in this paper performs quite well for low and moderate frequencies. In this section a set of representative numerical results are presented.

Consider the geometry in Fig. 7. The structure is illuminated by an externally generated wave $u^{inc}(x) = \exp \left( i\kappa x_3\right)$, where $\kappa$ is the wave number.

Results are displayed in Tables 1, 2, 3 and 4. For each reduction scheme, the columns ’unprec’ refer to the solution without preconditioner and the columns ’prec’ to the solution with preconditioner. In particular, Tables 1 and  3 summarise the GMRES iteration count for the Neumann and Dirichlet problem, respectively, at $\kappa =1.0$. Results are consistent with those for the Laplace problem ($\kappa =0$).

Tables 2 and 4 presents the GMRES iteration count for the Neumann and Dirichlet problem, respectively, at $\kappa =10.0$. Even though asymptotically preconditioning leads to a more efficient method, benefits in practice show up at much smaller values for *h*. For the Dirichlet problem in conjunction with the *single strip* reduction strategy, cross-over was not recorded within the range for *h* explored here.

**Table 1:** GMRES iterations at $\kappa =1.0$, Neumann problem

h	Full		Partial		Overlap		Strip	
	Unprec	Prec	Unprec	Prec	Unprec	Prec	Unprec	Prec
0.1	8	5	10	7	14	11	9	9
0.05	10	6	13	8	21	11	12	10
0.025	15	6	18	9	29	11	16	11
0.0125	21	7	26	9	41	12	21	12

**Table 2:** GMRES iterations at $\kappa =10.0$, Neumann problem

h	Full		Partial		Overlap		Strip	
	Unprec	Prec	Unprec	Prec	Unprec	Prec	Unprec	Prec
0.1	14	8	19	10	26	17	15	12
0.05	17	8	23	10	36	17	18	13
0.025	22	8	28	10	48	17	23	14
0.0125	28	9	37	12	66	18	29	16

**Table 3:** GMRES iterations at $\kappa =1.0$, Dirichlet problem

h	Full		Partial		Overlap		Strip	
	Unprec	Prec	Unprec	Prec	Unprec	Prec	Unprec	Prec
0.1	17	120	19	18	17	17	17	21
0.05	21	15	24	19	23	19	21	23
0.025	27	16	30	20	29	20	27	26
0.0125	34	17	37	22	35	21	34	30

**Table 4:** GMRES iterations at $\kappa =10.0$, Dirichlet problem

h	Full		Partial		Overlap		Strip	
	Unprec	Prec	Unprec	Prec	Unprec	Prec	Unprec	Prec
0.1	27	35	31	41	29	42	27	39
0.05	33	37	36	44	35	44	33	43
0.025	40	39	44	45	42	46	40	46
0.0125	48	39	53	47	50	46	48	49

## Conclusions

We have presented an effective Calderón-type preconditioner for potential problems in the exterior of multi-screens that builds on quotient-space BEM and operator preconditioning. Moreover, we have proved and confirmed numerically that it performs as standard Calderón preconditioning does on simple screens.

From a computational point of view, quotient-space BEM considering the full discretisation of multi-valued traces has the advantage of requiring minimal geometrical information but the disadvantage of *doubling the number of basis functions*. As an alternative, we proposed different strategies to work with reduced multi-trace discretisations that use less basis functions but require more adaptations when using a standard BEM code. We gave details regarding the additional data requirements in the implementation of all these strategies, and used the developed framework to identify the requirements that reduced spaces need to meet in order to still have efficient Calderón-type preconditioning.

Finally, we briefly presented a heuristic strategy to precondition multi-screens that also appear in applications but that are not covered by our theory. Although in essence our approach follows the same principles of our Calderón-type preconditioner for type A multi-screens, rigorous analysis has been elusive and therefore has not been treated in this article. Indeed, the key missing piece is an extension of Lemma 6 for this case. Nevertheless, we offered numerical experiments to investigate its effectivity.

Current and future work also involves extending the analysis of this preconditioning approach to Maxwell equations, where numerical results are promising [15], yet the construction of the jump aware projection $\textsf{R}_h$ is considerably more challenging.

## Footnotes

1 We remind the reader that $\textbf{H}(\textrm{div},\varOmega ):= \lbrace \textbf{v} \in L^2(\varOmega )^3\,: \, \textrm{div}\, \textbf{v} \in L^2(\varOmega )^3 \rbrace$ for $\varOmega =\mathbb {R}^3$ or $\varOmega =\mathbb {R}^3{\setminus }\overline{\varGamma }$, and refer to [19, Sect. 1.1] for definitions of the relevant Sobolev spaces.

2 Indeed, this definition of Neumann trace generalises the fact that for a Lipschitz domain $\varOmega \subset \mathbb {R}^3$, the normal derivative of $v \in H^1(\varDelta ,\varOmega )$ can be computed in $H^{- \frac{1}{2}} (\varOmega )$ by projecting $\nabla v\in \textbf{H}(\textrm{div},\varOmega )$ to the normal vector field $\textbf{n}$ of $\partial \varOmega$.

3 Expressions $\int \nolimits _{[\varGamma ]} \dots \ d\sigma$ should not be read as integrals with respect to the Lebesgue measure on $[\varGamma ]$. This is simply notation introduced in [9] and that we kept for later convenience.

4 Here we follow the numbering given by the underlying Lipschitz partition, while in Sect. 4.2 we let the user number $\mathcal {I}_l$ differently. This is harmless because one can always find a Lipschitz partition such that there is a one-to-one correspondence between $\mathcal {I}_l$ and $\varGamma _j$.

5 https://github.com/krcools/BEAST.jl.

6 https://github.com/krcools/Junctions_KC_CUT.jl.
